# Mn/Zn Bimetallic MOF‐Integrated Hydrogel Alleviates Excessive Mitochondrial Fission in BMSCs to Promote Osteoporotic Jawbone Repair

**DOI:** 10.1002/advs.78268

**Published:** 2026-10-15

**Authors:** Xiayue Jin, Shengjia Ye, Yun Yuan, Xueying Yang, Jiarui Cui, Hongming Zhang, Hui Huang

**Affiliations:** ^1^ Department of Prosthodontics Shanghai Ninth People's Hospital Shanghai Jiao Tong University School of Medicine Shanghai China; ^2^ College of Stomatology Shanghai Jiao Tong University Shanghai China; ^3^ National Center for Stomatology Shanghai China; ^4^ National Clinical Research Center for Oral Diseases Shanghai China; ^5^ Shanghai Key Laboratory of Stomatology Shanghai China; ^6^ Shanghai Research Institute of Stomatology Shanghai China; ^7^ Department of Stomatology Zhongshan Hospital Fudan University Shanghai China

**Keywords:** composite hydrogels, metal‐organic framework, mitochondrial fission, osteoporotic jawbone defect

## Abstract

Osteoporotic jawbone defects show impaired bone healing and insufficient new bone regeneration due to a pathological osteoporotic microenvironment, where chronic inflammation and excessive reactive oxygen species (ROS) accumulation impair the function of bone marrow‐derived mesenchymal stem cells (BMSCs). In this study, we find that osteoporotic BMSCs display reduced osteogenic differentiation and migration, accompanied by elevated intracellular ROS levels, mitochondrial dysregulation at both functional and structural levels. Targeting this pathophysiology, we engineer a locally injectable Mn‐ZIF‐8@GelMA hydrogel by incorporating manganese‐doped zeolitic imidazolate framework‐8 nanoparticles into a gelatin methacryloyl matrix, synergistically integrating redox regulation with osteogenic stimulation. Mn‐ZIF‐8@GelMA effectively alleviates oxidative stress, improves mitochondrial function, and the osteogenic competence of osteoporotic BMSCs. Mechanistically, Mn‐ZIF‐8@GelMA treatment increases mitochondrial antiviral signaling protein (MAVS) expression, reduces dynamin‐related protein 1 (DRP1) phosphorylation, and alleviates excessive mitochondrial fission. Mitochondrial disruption and MAVS knockdown further suggest that MAVS and DRP1 may contribute to the mitochondrial remodeling and osteogenic effects of Mn‐ZIF‐8@GelMA. In vivo, Mn‐ZIF‐8@GelMA significantly promotes extraction socket repair in osteoporotic mice. This work presents a Mn/Zn bimetallic metal‐organic framework (MOF)‐integrated GelMA hydrogel for osteoporotic jawbone repair, and highlights redox‐mediated mitochondrial regulation of endogenous BMSCs as a promising strategy to overcome impaired bone regeneration under osteoporotic conditions.

## Graphical abstract

Excessive reactive oxygen species (ROS) and chronic inflammation impair bone marrow‐derived mesenchymal stem cells (BMSCs), contributing to poor healing in osteoporotic jawbone defect. The gelatin methacryloyl hydrogel loaded with manganese‐doped zeolitic imidazolate framework (Mn‐ZIF‐8@GelMA) promotes regeneration through redox regulation and mitochondrial restoration, which is associated with increases mitochondrial antiviral signaling protein (MAVS) expression, reduced dynamin‐related protein 1 (DRP1) phosphorylation, and improved mitochondrial networks.

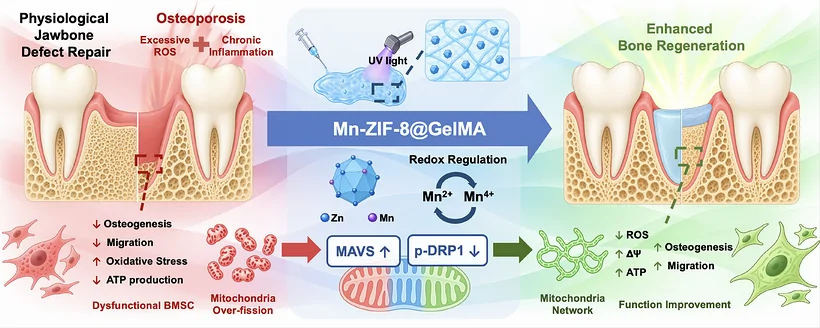

## 1 Introduction

Osteoporotic (OP) jawbone defects are characterized by impaired regenerative capacity, with tooth extraction representing the most common clinical scenario. Epidemiological studies show that reduced bone mineral density is associated with tooth loss [1, 2], while preclinical evidence consistently demonstrates delayed extraction socket repair under osteoporotic conditions [3]. Delayed and insufficient healing of jawbone defects compromises subsequent rehabilitation, thus impairing masticatory and phonatory oral function. Pathologically, delayed OP jawbone regeneration is closely linked to the imbalance between osteogenesis and osteoclast‐mediated bone resorption, coupled with a hostile local microenvironment characterized by chronic inflammation and acidic conditions. Alveolar ridge preservation (ARP) is commonly used to limit post‐extraction ridge reduction [4], but its effectiveness remains limited, as ARP procedures are highly technique‐sensitive, require strict case selection and soft‐tissue management, and are readily undermined by the osteoporotic microenvironment, which accelerates material loss and perpetuates impaired osteogenesis [5]. In parallel, systemic pharmacological therapies provide limited local bioavailability and, at high or prolonged doses, may increase the risk of medication‐related osteonecrosis of the jaw. These limitations reveal the need for therapeutic strategies that are locally deployable, clinically practical, and sufficiently robust to overcome OP‐driven regenerative resistance and rescue effective bone healing.

Biomaterials applied to extraction sockets have evolved from passive space‐maintaining grafts, such as bone substitute particles, collagen sponges, barrier membranes, and autologous blood‐derived products, toward multifunctional platforms [6]. Among them, hydrogels have attracted widespread attention due to their favorable biocompatibility, injectability, defect conformability, extracellular matrix‐like properties, and capacity for localized delivery of bioactive agents [5]. Currently, hydrogels used in bone tissue engineering often require the incorporation of growth factors such as bone morphogenetic protein 2 (BMP‐2) to achieve osteoinductivity [7]. However, their clinical application is limited by the need for doses far exceeding physiological levels, as well as the risks of local inflammation and ectopic bone formation [8, 9]. In addition, many composite hydrogel systems still face challenges such as uncertain long‐term biosafety, burst or poorly controlled release, possible off‐target biological effects, complex fabrication procedures, and high production costs [10]. In osteoporotic defects, these limitations may be further amplified by the impaired regenerative capacity of endogenous cells, suggesting that effective biomaterial strategies should not only provide osteogenic cues but also improve the intrinsic cellular competence for bone regeneration.

Bone marrow‐derived mesenchymal stem cells (BMSCs) are the principal cellular source for osteoblast lineage commitment and new bone formation. Accumulating evidence indicates that BMSCs derived from osteoporotic conditions exhibit intrinsic dysfunction, characterized by reduced cellularity, impaired proliferative capacity, and a lineage shift favoring adipogenesis over osteogenesis [11, 12, 13]. Dysfunction of BMSCs may limit the response to conventional biomaterial strategies. During osteogenic differentiation, MSCs undergo metabolic reprogramming from glycolysis to oxidative phosphorylation (OXPHOS), accompanied by increases in oxygen consumption rate and ATP production to meet the elevated energy demands [14]. Concurrently, intracellular reactive oxygen species (ROS) levels decline, alongside the upregulation of antioxidant defense systems, including superoxide dismutase (SOD), catalase (CAT), and glutathione peroxidase (GPx), thereby maintaining redox homeostasis. In contrast, BMSCs isolated from OP mice (OP‐mBMSCs) exhibit pronounced senescent phenotypes both in vitro and in vivo, accompanied by upregulation of senescence‐associated secretory phenotype (SASP) factors, which is closely associated with mitochondrial dysfunction [15]. Damaged mitochondria display impaired OXPHOS, during which excessive ROS are generated primarily from respiratory chain complexes I and III, thereby activating inflammatory and stress‐related signaling pathways such as AMPK and NF‐κB and releasing SASPs [16].

Recent studies find that excessive ROS accumulation induces dynamin‐related protein 1 (DRP1)‐dependent mitochondrial fission [17]. DRP1 is a member of the dynamin superfamily of GTPases. It is predominantly localized in the cytosol and is recruited to the mitochondrial outer membrane by adaptor proteins upon activation. Under oxidative stress conditions, such as H_2_O_2_ stimulation, increased DRP1 expression and phosphorylation are associated with mitochondrial fragmentation, swelling, and vesiculation, whereas inhibition or deficiency of DRP1 alleviates mitochondrial dysfunction and rescues cellular function [18]. However, the mechanistic relationship among ROS overproduction, DRP1‐mediated mitochondrial fission, and impaired osteogenic differentiation of BMSCs remains incompletely understood.

Targeting oxidative stress and restoring mitochondrial homeostasis in BMSCs may represent a promising strategy for improving bone regeneration under osteoporotic conditions. Zeolitic imidazolate framework‐8 (ZIF‐8), a zinc‐based metal‐organic framework (MOF), has been widely explored in bone tissue engineering due to its pH‐responsive degradation and sustained Zn^2+^ release. Our previous studies have demonstrated that ZIF‐8 enhances osseointegration of porous titanium implants under diabetic inflammatory conditions, highlighting its therapeutic potential in pathological microenvironments [19]. However, ZIF‐8 lacks intrinsic redox activity, limiting its effectiveness in OP bone defects. Manganese (Mn), an essential trace element, can serve as a cofactor for manganese superoxide dismutase and accumulates in mitochondria, where it detoxifies reactive oxygen species and mitigates oxidative stress [20]. The antioxidant capability of Mn is attributed to its multiple valence states. For example, Mn^2+^ donates electrons to neutralize superoxide anions (•O_2_^−^), while Mn^3+^ catalyzes the decomposition of hydrogen peroxide (H_2_O_2_) [21]. This redox cycling endows Mn‐based systems with enzyme‐mimicking antioxidant capabilities, making them particularly suitable for addressing oxidative stress‐dominated pathological conditions [22].

In summary, we designed a Mn‐incorporated ZIF‐8 bimetallic MOF and further integrated it into a hydrogel matrix to construct a locally applicable composite system with combined osteogenic and antioxidative functions. This novel composite hydrogel represents a bioactive material strategy capable of converting an osteoporosis‐compromised socket to a pro‐regenerative one by modulating the local oxidative microenvironment and restoring the mitochondrial function of endogenous BMSCs. Our study explored the ROS‐mitochondrial fission‐cellular homeostasis axis, with a particular focus on whether excessive oxidative stress promotes DRP1‐mediated mitochondrial fission and thereby impairs the osteogenic competence of BMSCs under osteoporotic conditions.

## 2 Results and Discussion

### 2.1 Osteoporotic Conditions Impair BMSC Function and Disrupt Mitochondrial Homeostasis

#### 2.1.1 Impaired Osteogenic Differentiation and Oxidative Stress in OP‐mBMSCs

In the OP mouse model, bone volume loss and microstructure deterioration were confirmed in both the femur and jawbone (Figures S1C and S2A). OP‐mBMSCs exhibited markedly reduced mineralized nodule formation and increased lipid droplet accumulation in vitro (Figure 1A), confirming their multilineage differentiation potential and indicating a concomitant shift toward adipogenic lineage commitment under OP conditions. Osteogenic differentiation of MSCs is regulated by multiple signaling pathways, including bone morphogenetic proteins (BMPs), Wnt, and Notch pathways, among which BMP‐2 plays a central role by activating key transcription factors such as Runt‐related transcription factor 2 (RUNX2) and Osterix (OSX) [23, 24, 25]. Consistently, the expression levels of osteogenesis‐related genes (*alpl, runx2, bmp2*, and *col1a1*) and the protein expression of BMP‐2 were significantly reduced in OP‐mBMSCs (Figure 1B,C).

**FIGURE 1:**
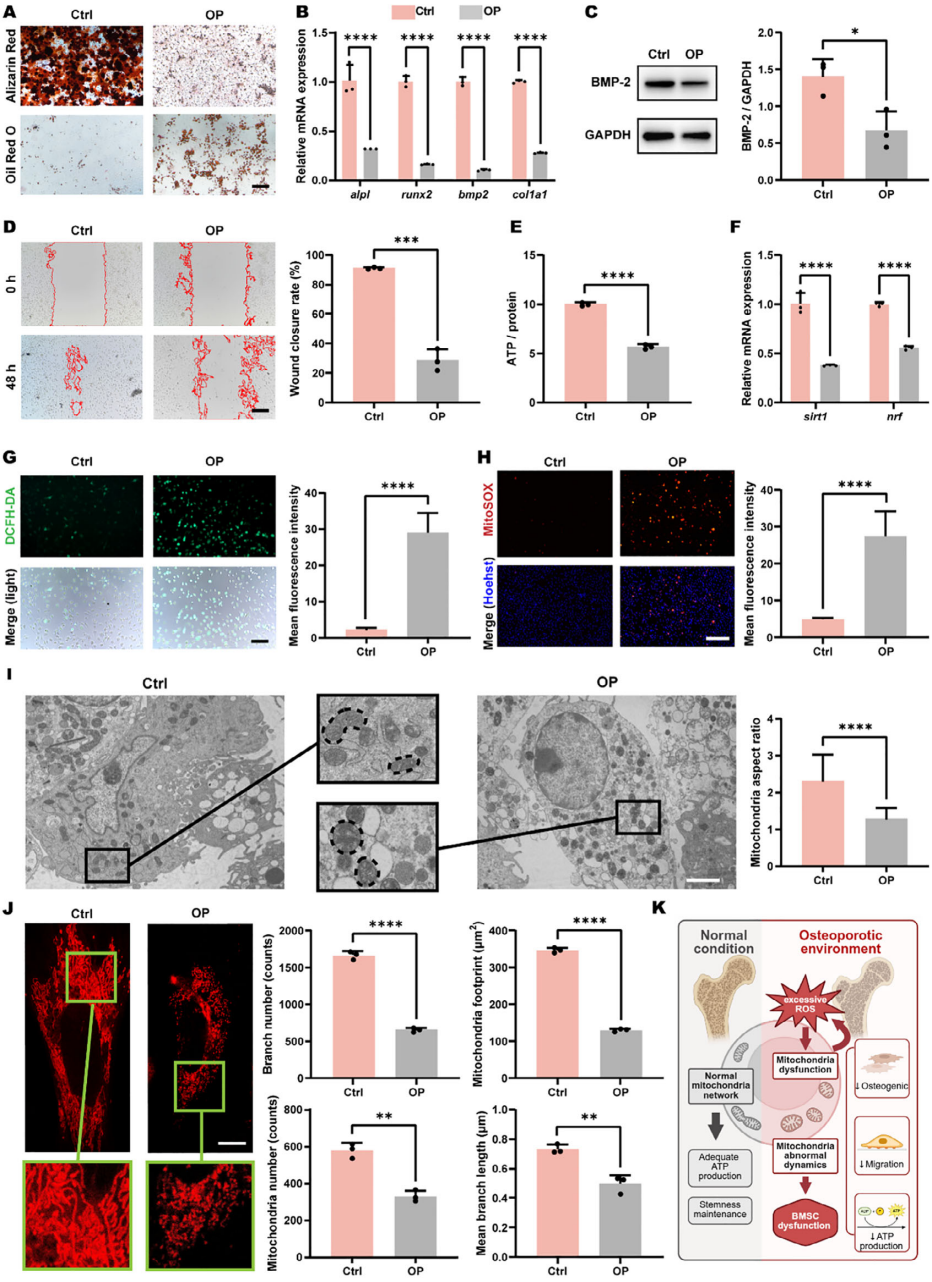
OP‐mBMSCs show impaired osteogenesis and migration accompanied by oxidative stress and mitochondrial dysfunction. (A) Representative optical images of Alizarin Red staining and Oil Red O staining illustrating osteogenic and adipogenic differentiation capacities of mBMSCs (*n* = 3 each group). Scale bar: 500 µm. (B) Relative mRNA expression levels of osteogenic‐related genes (*alpl, runx2, bmp2, col1a1*) (*n* = 3 each group). (C) Western blotting of BMP‐2 protein expression and quantitative analysis of gray value (*n* = 3 each group). (D) Representative optical images of the scratch wound assay illustrating the migration capacities of mBMSCs at 0 h and 48 h after scratching, with quantitative analysis of the wound closure rate (*n* = 3 each group). Scale bar: 500 µm. (E) Quantitative analysis of ATP production normalized to total protein content (*n* = 3 each group). (F) Relative mRNA expression levels of antioxidant‐related genes *nrf2* and *sirt1* (*n* = 3 each group). (G) Representative fluorescence images of intracellular ROS detected by DCFH‐DA and quantitative analysis (*n* = 3 each group). Scale bar: 200 µm. (H) Representative fluorescence images of mitochondrial ROS detected by MitoSOX and quantitative analysis (*n* = 3 each group). Scale bar: 500 µm. (I) Representative TEM images showing mitochondrial ultrastructure and quantitative analysis of the mitochondrial aspect ratio. Scale bar: 2 µm. (J) Representative confocal microscopy images of mitochondrial networks. Scale bar:10 µm. Magnified regions highlight detailed mitochondrial morphology changes. Quantitative analyses of mitochondria number, mean branch length, branch number, and mitochondrial footprint area showed significantly impaired mitochondrial networks in OP‐mBMSCs (*n* = 3 each group). (K) Schematic illustration summarizing the association among excessive ROS accumulation, mitochondrial dysfunction, impaired mitochondrial dynamics, and impaired osteogenic differentiation, migration, and ATP production in OP‐mBMSCs. Error bars indicate the mean ± SD. ^**^ *p* < 0.01, ^***^ *p* < 0.001, ^****^ *p* < 0.0001.

Effective stem cell recruitment to bone defects and infiltration into biomaterials are closely dependent on cellular migratory capacity [26]. Scratch wound assays showed that OP‐mBMSCs exhibited a significantly lower wound closure rate than normal mBMSCs (Figure 1D). Impaired migration may compromise endogenous cell recruitment and the efficacy of biomaterial‐mediated bone regeneration. Given that both osteogenic differentiation and migration are energy‐demanding processes, we subsequently assessed cellular ATP production. OP‐mBMSCs showed significantly less ATP production, indicating impaired cellular bioenergetic activity (Figure 1E).

Mitochondria are closely involved in cellular energy production and can both generate ROS and be damaged by sustained oxidative stress. The concomitant impairment of migration and ATP production suggested that the functional decline of OP‐mBMSCs might be associated with disrupted mitochondrial metabolism and redox homeostasis, so we next evaluated the oxidative stress status of OP‐mBMSCs. The expression of key antioxidant genes (*sirt1* and *nrf2*) was significantly decreased in OP‐mBMSCs (Figure 1F). Sirtuin1 (SIRT1) and nuclear factor erythroid 2‐related factor 2‌ (NRF2) serve as critical regulators of cellular antioxidant defense and mitochondrial homeostasis. SIRT1 enhances cellular resistance to oxidative stress during osteogenesis [27], whereas activation of the NRF2‐associated signaling reduces ROS accumulation and mitochondrial damage [28]. Consistent with the reduced antioxidant response, increased total intracellular ROS levels and a marked accumulation of mitochondrial ROS were observed in OP‐mBMSCs (Figure 1G,H), indicating that the oxidative imbalance in OP‐mBMSCs involves both an overall increase in intracellular ROS and a substantial mitochondrial ROS component.

The impaired migration capacity, the reduced ATP level, together with the accumulation of cellular and mitochondrial ROS, suggested that mitochondrial function was compromised in OP‐mBMSCs. Mitochondrial dysfunction may promote excessive mitochondrial ROS generation, whereas sustained oxidative stress from both mitochondrial and non‐mitochondrial sources may further impair mitochondrial function and disturb mitochondrial dynamics. These processes may therefore reinforce one another and contribute to the persistent functional impairment of OP‐mBMSCs. Since the maintenance of mitochondrial structure and network organization provides an important structural basis for efficient energy metabolism and redox homeostasis, we next examined mitochondrial morphology to determine whether the reduced ATP production level and increased mitochondrial ROS were accompanied by structural alterations.

#### 2.1.2 Mitochondrial Morphological Abnormalities in OP‐mBMSCs

Mitochondrial morphology is dynamically maintained through the coordinated processes of fusion and fission [29]. Through these dynamic processes, mitochondria can adopt a wide range of structural configurations, from discrete spherical forms to highly interconnected networks [30]. Alterations in mitochondrial morphology and network organization are closely associated with changes in cellular metabolism and pathological conditions [31]. Consistent with this notion, mitochondrial ultrastructural alterations were observed in OP‐mBMSCs (Figure 1I). In control mBMSCs, mitochondria displayed relatively regular morphology, typically appearing as short rods or small tubules with intact outer membranes and well‐defined, densely and orderly arranged cristae. In contrast, OP‐mBMSCs exhibited markedly swollen mitochondria that were predominantly rounded, with blurred and fragmented cristae and disorganized matrix electron density. Quantitative analysis further showed a significant reduction in the mitochondrial aspect ratio in OP‐mBMSCs, consistent with a shift from elongated to more rounded mitochondrial morphology.

At the network level, mitochondrial organization was also markedly altered (Figure 1J). Control mBMSCs showed elongated mitochondria widely distributed throughout the cytoplasm, forming an interconnected mitochondrial network, whereas OP‐mBMSCs exhibited punctate mitochondria that were enriched in the perinuclear region. Quantitative morphometric analysis further demonstrated significant reductions in mitochondrial number, branch number, mean branch length, and mitochondrial footprint area in OP‐mBMSCs. These structural and network alterations are widely recognized as hallmarks of excessive mitochondrial fission and collapse of the mitochondrial network [32]. Since mitochondrial integrity is essential for maintaining cellular bioenergetics and supporting BMSC differentiation, these abnormalities may partly explain the impaired osteogenic and migratory capacities of OP‐mBMSCs. Thus, effective biomaterial intervention in osteoporotic bone defects may require not only osteo‐inductive activity but also the capacity to re‐establish mitochondrial homeostasis at the cellular level.

### 2.2 Characterization of Mn‐ZIF‐8 and Mn‐ZIF‐8@GelMA

#### 2.2.1 Physicochemical Properties

We next sought to construct a bioactive material system capable of simultaneously providing structural support and modulating the cellular microenvironment. Previous studies on multi‐metallic ZIFs have shown that metal‐node engineering can modulate local electronic structure, defect distribution, and framework stability, thereby generating properties not achievable in monometallic systems [33]. In our study, Mn was introduced into the ZIF‐8 structure to construct a bimetallic MOF, Mn‐ZIF‐8. The synthesis process of the material is illustrated in Figure 2A. Zn^2+^/Mn^2+^ can be co‐precipitated with 2‐methylimidazole to form structurally compatible tetrahedral nodes because both divalent metal ions can coordinate with imidazolate linkers and co‐assemble within a ZIF‐8‐like framework [34, 35].

**FIGURE 2:**
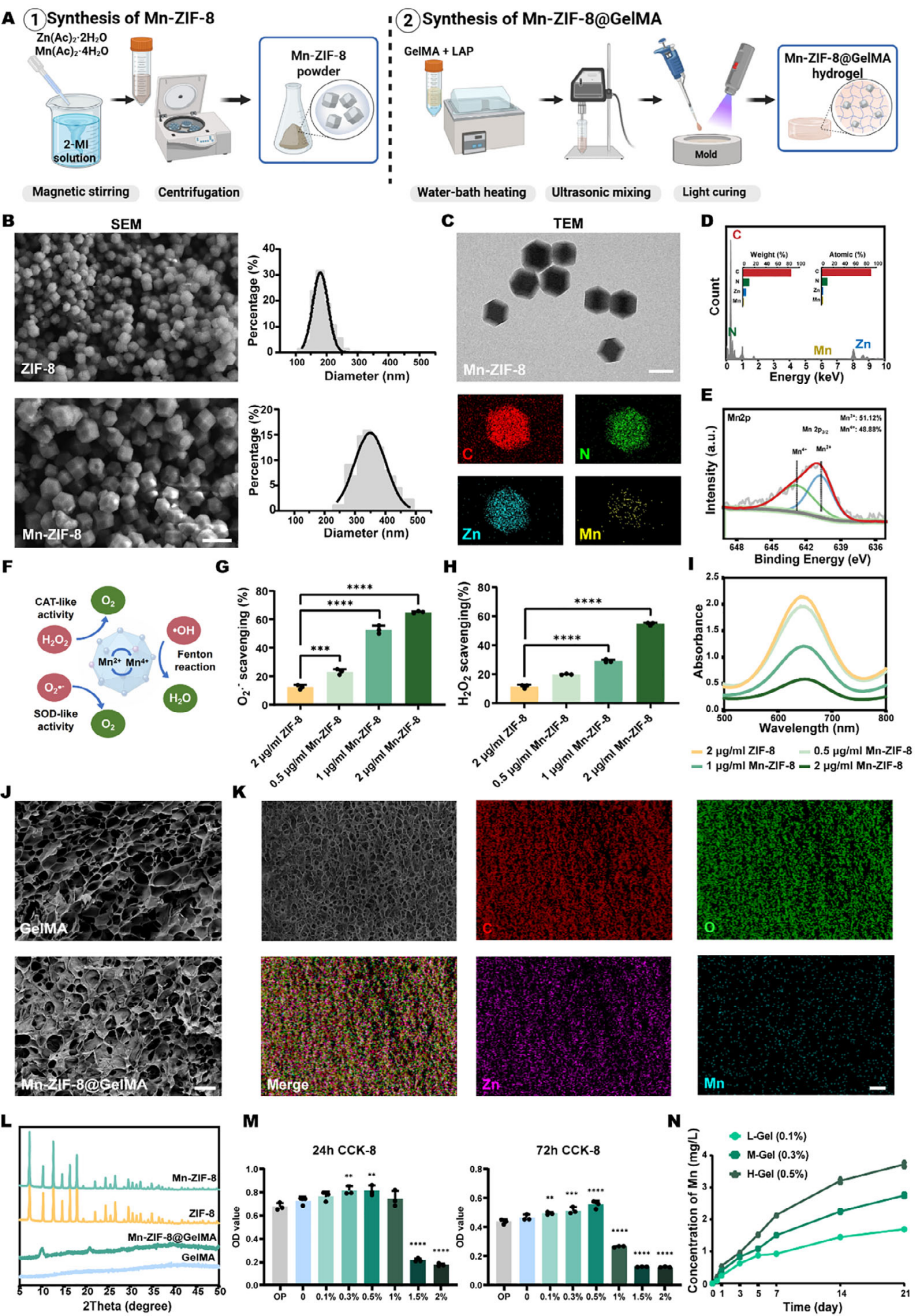
Synthesis and characterizations of Mn‐ZIF‐8 and Mn‐ZIF‐8@GelMA. (A) Schematic illustration of the synthesis procedures. (B) Representative SEM images and size distribution analysis of ZIF‐8 and Mn‐ZIF‐8 nanoparticles. Scale bar: 500 nm. (C) TEM images and (D) EDS elemental mapping of Mn‐ZIF‐8 nanoparticles. Scale bar: 500 nm. (E) XPS spectra demonstrating Mn2p valence state distribution (Mn^2+^ and Mn^4+^) in Mn‐ZIF‐8 nanoparticles. (F) Schematic illustration of the multienzyme‐like reactive oxygen species‐scavenging activities of Mn‐ZIF‐8. (G) H_2_O_2_‐scavenging activity and (H) •O_2_^−^‐scavenging activity of ZIF‐8 and Mn‐ZIF‐8 at the indicated concentrations (*n* = 3 each group). (I) UV–vis absorption spectra of the Fenton‐TMB reaction system after treatment with ZIF‐8 or Mn‐ZIF‐8 at the indicated concentrations, reflecting •OH‐scavenging activity (*n* = 3 each group). (J) SEM images displaying the porous microstructure of GelMA and Mn‐ZIF‐8@GelMA hydrogels. Scale bar: 100 µm. (K) SEM image and corresponding EDS elemental mappings illustrating homogeneous distribution of elements within Mn‐ZIF‐8@GelMA. Scale bar: 100 µm. (L) XRD patterns confirming the crystalline structures of ZIF‐8, Mn‐ZIF‐8, GelMA and Mn‐ZIF‐8@GelMA. (M) CCK‐8 showing the biocompatibility of Mn‐ZIF‐8@GelMA hydrogels at different concentrations after 24 and 72 h incubation (*n* = 3 each group). (N) Cumulative Mn ion release profiles from Mn‐ZIF‐8@GelMA hydrogels (L‐Gel, M‐Gel, H‐Gel) over 21 days (*n* = 3 each group). Error bars indicate the mean ± SD. ^*^ *p* < 0.05, ^**^ *p* < 0.01, ^***^ *p* < 0.001, ^****^ *p* < 0.0001.

The synthesized Mn‐ZIF‐8 maintained the typical morphology of ZIF‐8, presenting a regular rhombic dodecahedral structure, but with a larger particle size (Figure 2B). The average diameter of Mn‐ZIF‐8 was 348.83 ± 56.98 nm, compared with 186.09 ± 26.75 nm for ZIF‐8. This increase in particle size is attributed to the incorporation of Mn ions, which slows the nucleation process and reduces the number of nucleation centers, allowing larger nanoparticles to grow [36]. Elemental mapping further demonstrated a homogeneous distribution of C, N, Zn, and Mn throughout the particles (Figure 2C), supporting successful incorporation of Mn into the framework rather than simple surface contamination or phase segregation. The actual Mn content accounted for 0.04% of the total metal elements (Figure 2D). XPS was performed to analyze the valence states of Mn in Mn‐ZIF‐8 (Figure 2E). After calibration using the C 1s binding energy (284.7 eV), the Mn 2p_3/2_ spectrum showed a main peak between approximately 639 and 642 eV. Peak deconvolution revealed binding energies at ∼640 eV corresponding to Mn^2+^ and at ∼642 eV corresponding to Mn^4+^, accounting for 51.12% and 48.88% of the total peak area, respectively. The coexistence of variable Mn valence states and their uniform distribution indicates that Mn‐ZIF‐8 possesses a redox‐active metal‐node environment rather than a single‐valence doped state. In mixed‐metal MOFs, introducing a second metal center is known to perturb the local electronic structure, generate additional heterometallic active sites, and facilitate intersite electron transfer, thereby endowing the framework with properties that are often inaccessible to monometallic analogues [37]. From this perspective, the mixed‐valence Mn species observed here likely provide a structural basis for enhanced redox responsiveness, which is particularly relevant for subsequent ROS‐regulatory applications [38]. To further verify its intrinsic antioxidant capability, we evaluated the ROS‐scavenging performance of Mn‐ZIF‐8 in vitro (Figure 2F). Mn‐ZIF‐8 exhibited concentration‐dependent scavenging of •O_2_^−^, H_2_O_2_, and •OH, whereas ZIF‐8 showed only limited activity under the same conditions (Figure 2G–I). These results indicate that Mn incorporation endowed Mn‐ZIF‐8 with multiple ROS‐scavenging activities, including SOD‐like, CAT‐like, and •OH‐scavenging effects.

To enable the local delivery and retention of Mn‐ZIF‐8 within irregular extraction sockets, Gelatin methacryloyl (GelMA) was subsequently employed as the hydrogel matrix. GelMA has been widely used in bone tissue engineering because it combines biocompatibility, photocrosslinkability, injectability, cell‐adhesive motifs, and a hydrated matrix resembling extracellular matrix. To provide a permissive matrix for tissue ingrowth and allow local adaptation to the irregular geometry of extraction sockets, GelMA was used as a hydrogel matrix with Mn‐ZIF‐8 incorporated to create composite hydrogel. The Mn‐ZIF‐8@GelMA hydrogel precursor showed good fluidity, allowing minimally invasive administration and conformal adaptation to irregular defects. Following brief ultraviolet irradiation, it rapidly underwent in situ crosslinking and solidification, resulting in a stable three‐dimensional structure with good shape stability and adhesive properties (Figure S3). Mn‐ZIF‐8@GelMA possessed a porous three‐dimensional network structure similar to that of GelMA, providing a favorable microenvironment for cell infiltration, nutrient and oxygen diffusion, and metabolic waste exchange (Figure 2J). Oxygen (O) from the GelMA matrix, together with zinc (Zn) and manganese (Mn) from Mn‐ZIF‐8, were uniformly distributed throughout the three‐dimensional network, indicating successful incorporation of Mn‐ZIF‐8 nanoparticles without obvious aggregation (Figure 2K). The XRD pattern of Mn‐ZIF‐8 exhibited the characteristic diffraction peaks of ZIF‐8, indicating that the crystal structure retained the typical ZIF‐8 topology, while slight variations in peak positions and intensities were observed, which may be attributed to lattice distortion induced by Mn doping (Figure 2L). The XRD pattern of Mn‐ZIF‐8@GelMA showed broad and weak diffraction peaks consistent with the amorphous nature of GelMA, while the characteristic peaks of Mn‐ZIF‐8 became weaker and broader, likely due to the encapsulation effect of the GelMA matrix (Figure 2L). The compressive and tensile moduli of Mn‐ZIF‐8@GelMA increased gradually with increasing nanoparticle concentration (Figure S4), suggesting that the nanoparticles may participate in the hydrogel polymer network and provide additional physical anchoring sites. In summary, the composite hydrogel allowed direct in situ application with rapid on‐site gelation, providing convenient operability and mechanical properties suitable for jawbone defect sites.

#### 2.2.2 In Vitro Cytocompatibility and Ion Releasing Profile

The cytocompatibility of Mn‐ZIF‐8@GelMA was further evaluated. After 24 h, hydrogels containing more than 0.5% Mn‐ZIF‐8 exhibited certain cytotoxic effects. After 72 h of co‐culture, hydrogels containing 0.1%, 0.3%, and 0.5% Mn‐ZIF‐8 exhibited significantly increased OD values (Figure 2M), indicating good cytocompatibility and a promotive effect on cell proliferation within this concentration range. These findings suggest that appropriate incorporation of Mn‐ZIF‐8 not only avoids obvious cytotoxicity, but may also provide a permissive microenvironment for cell survival and proliferation.

The biological activity of the composite hydrogel is closely associated with the concentration of released Mn ions. To simulate the degradation process of the composite hydrogel in the osteoporotic jawbone defect microenvironment, hydrogels from different groups were immersed in buffer solution, and the concentration of released Mn ions was monitored over time. The composite hydrogels degraded gradually and continuously released Mn ions (Figure 2N). After 21 days, the hydrogels were completely degraded. At this time point, the Mn concentration released from hydrogels containing 0.1% (w/v) Mn‐ZIF‐8 (L‐Gel), 0.3% (w/v) Mn‐ZIF‐8 (M‐Gel), and 0.5% (w/v) Mn‐ZIF‐8 (H‐Gel) reached 1695.96 ± 7.83, 2748.36 ± 31.28, and 3719.79 ± 72.16 µg/L, respectively. The release profile of Zn^2^^+^ from ZIF‐8@GelMA and the corresponding Mn/Zn release from Mn‐ZIF‐8@GelMA were further characterized (Figure S5). These results indicate that Zn and Mn ion release from the composite hydrogel is concentration‐dependent and sustained throughout the degradation process. The sustained and dose‐dependent ion release observed here suggests that Mn‐ZIF‐8@GelMA can serve as a local reservoir of bioactive ions, thereby providing persistent biochemical support for redox regulation and cellular activities during the repair process.

While Mn ion release represents an important pathway for the biological activity of Mn‐ZIF‐8@GelMA, whether Mn‐ZIF‐8 nanoparticles themselves could be internalized by cells remained unclear. Electron‐dense nanoscale structures were observed within cells after Mn‐ZIF‐8@GelMA treatment. Some particles retained relatively intact polyhedral morphologies resembling the original rhombic dodecahedral structure of Mn‐ZIF‐8, whereas others exhibited blurred boundaries, irregular shapes, or partial structural collapse (Figure S6). These observations suggest that at least a fraction of Mn‐ZIF‐8 nanoparticles can be internalized by cells and undergo intracellular structural transformation. Together with the sustained ion release profile, these findings indicate that Mn‐ZIF‐8@GelMA may exert biological effects through multiple forms. However, the relative contribution of these pathways and the precise intracellular fate of Mn and Zn ions require further investigation.

#### 2.2.3 Antibacterial Activity of Mn‐ZIF‐8@GelMA

Antibacterial biomaterials may provide additional protection against infection during socket regeneration [39, 40]. Mn‐ZIF‐8@GelMA exhibited evident antibacterial activity against both *Escherichia coli* and *Streptococcus mutans* (Figure 3). Moreover, the antibacterial efficacy increased with increasing Mn‐ZIF‐8 content, demonstrating a clear concentration‐dependent effect. These findings suggest that incorporation of Mn‐ZIF‐8 substantially enhances the antibacterial performance of GelMA hydrogel and enables effective suppression of both Gram‐negative and Gram‐positive bacteria. Such broad‐spectrum activity is consistent with previous reports showing that ZIF‐8‐based and metal ion‐releasing biomaterials can inhibit bacterial growth through membrane disruption, oxidative stress induction, and local ion‐mediated antibacterial effects [41]. Given that bacterial colonization is a major challenge in the oral environment, the antibacterial property of Mn‐ZIF‐8@GelMA may be beneficial for reducing early bacterial adhesion and lowering the risk of infection during socket healing, thereby creating a more favorable microenvironment for subsequent tissue regeneration [6].

**FIGURE 3:**
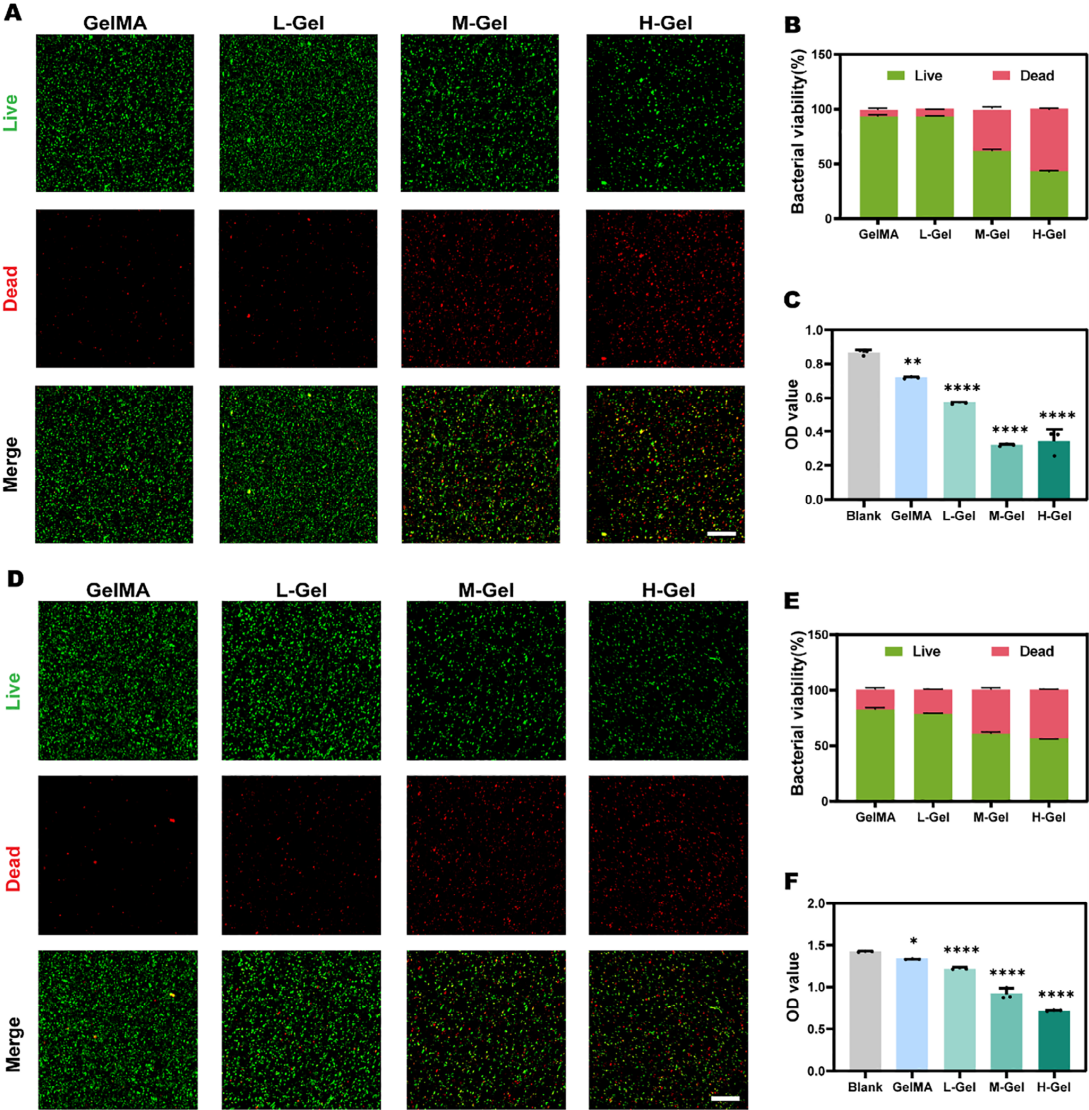
Antibacterial evaluation of Mn‐ZIF‐8@GelMA hydrogels. Live/Dead staining images of (A) *E. coli* and (D) *S. mutans* cultured on different hydrogels (*n* = 3 each group). Scale bar: 100 µm. Green indicates live bacteria, and red indicates dead bacteria, with the proportion of dead cells increasing as Mn‐ZIF‐8 concentration increased. Quantitative analysis of bacterial viability of (B) *E. coli* and (E) *S. mutans* in each group (*n* = 3 each group). OD600 values of (C) *E. coli* and (F) *S. mutans* showing bacterial growth inhibition with increasing Mn‐ZIF‐8 content (*n* = 3 each group). Error bars indicate the mean ± SD. ^*^ *p* < 0.05, ^**^ *p* < 0.01, ^***^ *p* < 0.001, ^****^ *p* < 0.0001.

#### 2.2.4 Hemostatic Performance of Mn‐ZIF‐8@GelMA

Beyond infection control, rapid hemostasis and stabilization of the blood clot are also critical for favorable extraction socket healing. In the mouse liver injury model, Mn‐ZIF‐8@GelMA markedly reduced blood loss compared with the untreated control, indicating that the hydrogel possessed effective hemostatic capability (Figure S7). Compared with the untreated group (Blank), application of the hydrogel significantly reduced bleeding. Although no statistically significant differences were observed among the hydrogel groups, the M‐Gel and H‐Gel groups tended to exhibit lower blood loss. Notably, GelMA without nanoparticle loading also demonstrated favorable hemostatic performance, which may be attributed to the intrinsic adhesive and absorptive properties of the hydrogel matrix [42]. These findings suggest that the hydrogel possesses advantageous hemostatic properties and may have practical potential for postoperative bleeding control and wound healing, making it suitable as a sealing material for extraction sockets [43].

### 2.3 Biological Effects of Mn‐ZIF‐8@GelMA on OP‐mBMSCs In Vitro

#### 2.3.1 Mn‐ZIF‐8@GelMA Promotes Osteogenic Differentiation

As shown in the previous section, OP‐mBMSCs exhibited a markedly impaired osteogenic phenotype. In contrast, Mn‐ZIF‐8@GelMA substantially reversed this dysfunction, as evidenced by enhanced mineralized nodule formation and reduced lipid droplet accumulation, with the M‐Gel group showing the strongest effect (Figure 4A). This result suggests that Mn‐ZIF‐8@GelMA partially rebalanced osteogenic‐adipogenic lineage commitment of OP‐mBMSCs. Consistent with these phenotypic changes, Mn‐ZIF‐8@GelMA significantly upregulated the expression of osteogenesis‐related genes, including *alpl, runx2, bmp2*, and *col1a1*, and also increased the protein level of BMP‐2, with the M‐Gel group exhibiting the most pronounced upregulation (Figure 4B,C). BMP‐2 is not merely a downstream osteogenic marker, but a central osteo‐inductive factor capable of initiating and amplifying osteogenic signaling [44]. More importantly, emerging evidence indicates that BMP‐2 is closely linked to mitochondrial regulation during osteogenesis [45, 46]. BMP‐2 can promote mitochondrial activity and biogenesis‐related signaling, thereby coupling osteogenic commitment to the metabolic adaptation required for matrix production and mineralization [47]. Therefore, the increase in BMP‐2 expression observed here is meaningful not only as evidence of enhanced osteogenesis, but also as an early indication that Mn‐ZIF‐8@GelMA may improve the mitochondrial state of OP‐mBMSCs.

**FIGURE 4:**
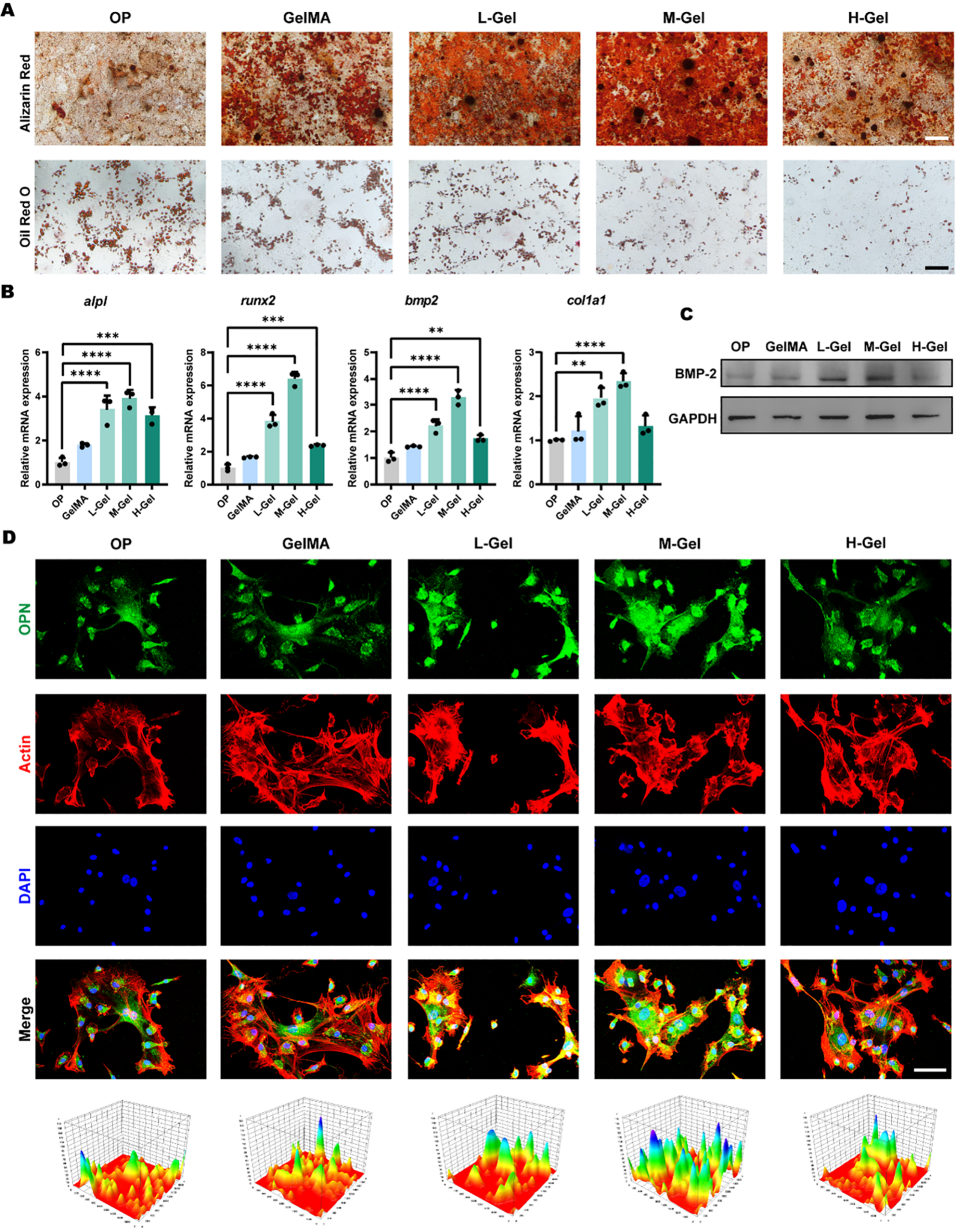
Mn‐ZIF‐8@GelMA hydrogels promote osteogenic differentiation of OP‐mBMSCs in vitro. (A) Representative images of Alizarin Red S and Oil Red O staining showing enhanced mineralized nodule formation and reduced lipid droplet accumulation (*n* = 3 each group). Scale bar: 500 µm. (B) Quantitative real‐time PCR analysis of osteogenic‐related genes (*alpl, runx2*, *bmp2*, and *col1a1*) in different groups (*n* = 3 each group). (C) Western blot analysis of BMP2 protein expression, with GAPDH as an internal control (*n* = 3 each group). (D) Immunofluorescence staining of OPN (green), actin cytoskeleton (red), and nuclei (DAPI, blue), with merged images and 3D reconstruction of fluorescence intensity maps (*n* = 3 each group). Scale bar: 50 µm. Error bars indicate the mean ± SD. ^*^ *p* < 0.05, ^**^ *p* < 0.01, ^***^ *p* < 0.001, ^****^ *p* < 0.0001.

Immunofluorescence staining showed that OP‐mBMSCs cultured with Mn‐ZIF‐8@GelMA displayed a more spread morphology and well‐organized cytoskeleton, accompanied by significantly increased osteopontin (OPN) expression. OPN is a late osteogenic marker associated with osteoblast maturation, matrix remodeling and cell‐matrix interaction [48]. OPN can be expressed through the BMP pathway and participates in integrin‐mediated signaling and cytoskeletal organization during MSC osteogenesis [49, 50]. Cell spreading and cytoskeletal organization are closely coupled to fate determination in mesenchymal stem cells, and a well‐developed actin cytoskeleton is generally considered more permissive for osteogenic differentiation [51]. In this context, the concurrent increase in OPN expression and cytoskeletal organization suggests that Mn‐ZIF‐8@GelMA rescued a more osteogenesis‐permissive cellular architecture in OP‐mBMSCs. Among the tested formulations, the M‐Gel group showed the most pronounced cytoskeletal remodeling and osteogenic protein expression (Figure 4D). Comparison with normal mBMSCs showed that Mn‐ZIF‐8@GelMA promoted the recovery of OP‐mBMSC osteogenic capacity toward a physiological state (Figure S8).

Taken together, these findings indicate that Mn‐ZIF‐8@GelMA rescues osteogenic differentiation of OP‐mBMSCs in a coordinated manner, encompassing lineage commitment, matrix synthesis, and osteogenesis‐associated cellular architecture. Since these processes are highly energy‐dependent and tightly regulated by mitochondrial homeostasis, the robust recovery of osteogenic capacity observed here strongly suggests that mitochondrial dysfunction may be an upstream constraint on OP‐mBMSC fate.

#### 2.3.2 Mn‐ZIF‐8@GelMA Alleviates Mitochondrial Dysfunction and Oxidative Stress in OP‐mBMSCs

Given the central role of mitochondrial homeostasis in regulating stem cell fate and osteogenic differentiation, we next investigated whether mitochondrial function recovery contributes to the pro‐osteogenic effect of Mn‐ZIF‐8@GelMA in OP‐mBMSCs. Mn‐ZIF‐8@GelMA treatment markedly reduced ROS levels, with the most pronounced effect observed in the M‐Gel group (Figure 5A). OP‐mBMSCs in the OP group displayed mitochondrial depolarization and functional impairment, while Mn‐ZIF‐8@GelMA significantly rescued mitochondrial membrane potential, indicating improved mitochondrial integrity (Figure 5B). Because mitochondrial membrane potential is a key indicator of mitochondrial integrity and bioenergetic activity, its recovery strongly suggests that Mn‐ZIF‐8@GelMA alleviated mitochondrial dysfunction rather than merely reducing intracellular oxidative burden [52]. Treatment with L‐Gel and M‐Gel significantly increased ATP levels compared with the OP and GelMA groups, with the M‐Gel group exhibiting the highest ATP production (Figure 5C).

**FIGURE 5:**
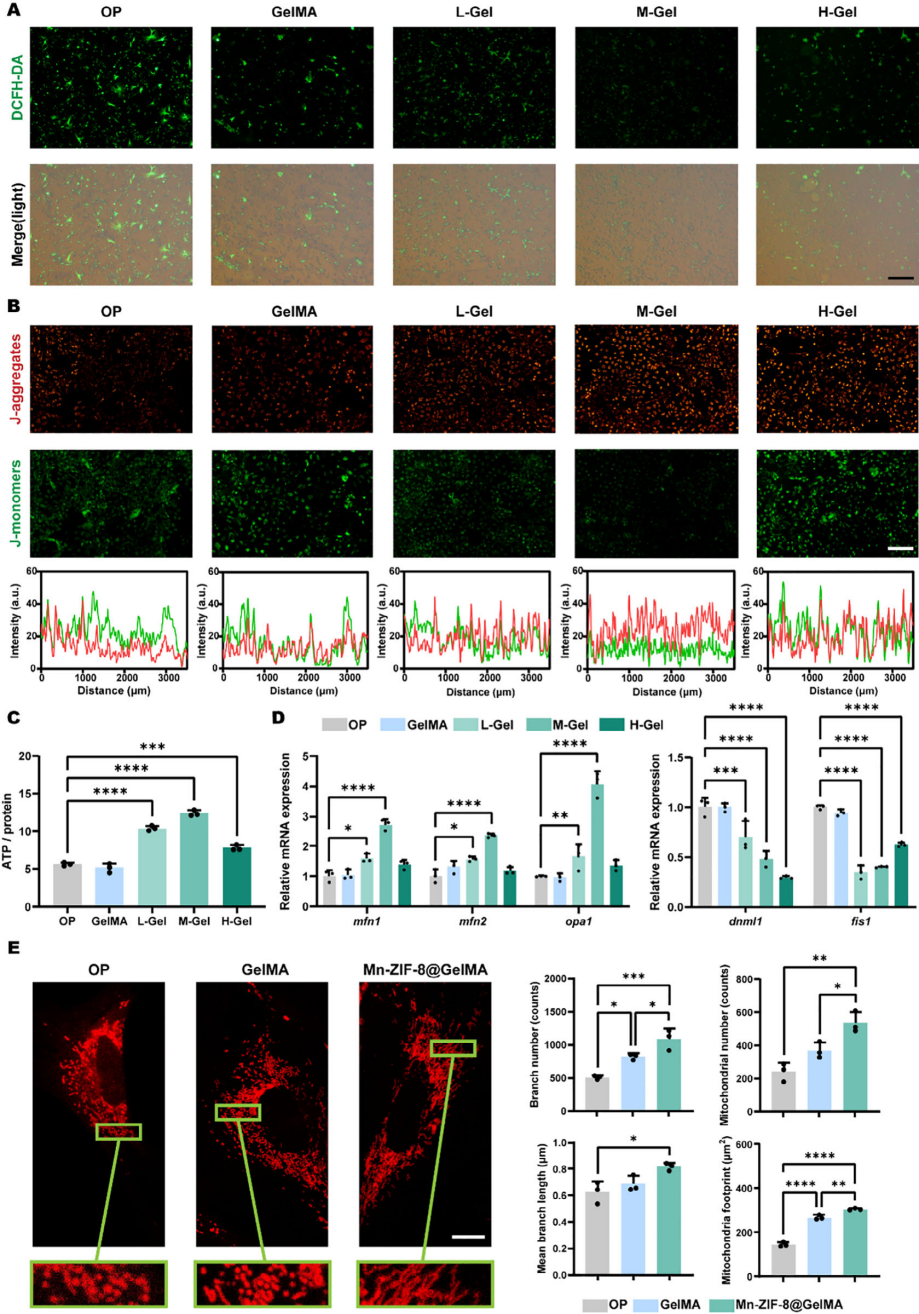
Mn‐ZIF‐8@GelMA alleviates oxidative stress and rescues mitochondrial dynamics in OP‐mBMSCs. (A) Representative fluorescence images of intracellular ROS levels detected by DCFH‐DA staining in different groups (*n* = 3 each group). Scale bar: 500 µm. (B) JC‐1 staining showing mitochondrial membrane potential, with red fluorescence indicating J‐aggregates (normal potential) and green fluorescence indicating J‐monomers (depolarized mitochondria) (*n* = 3 each group). Scale bar: 500 µm. Corresponding fluorescence intensity profiles are shown below. (C) Intracellular ATP content measured in OP‐mBMSCs cultured with different hydrogels (*n* = 3 each group). (D) Relative mRNA expression levels of mitochondrial dynamics–related genes, including the fusion‐associated genes *mfn1*, *mfn2*, and *opa1* and the fission‐associated genes *dnm1l* and *fis1*, in each group (*n* = 3 each group). (E) Mitochondrial morphology visualized by confocal microscopy and quantitative analysis of mitochondrial number, branch number, branch length, and mitochondrial footprint (*n* = 3 each group). Scale bar: 10 µm. Error bars indicate the mean ± SD. ^*^ *p* < 0.05, ^**^ *p* < 0.01, ^***^ *p* < 0.001, ^****^ *p* < 0.0001.

Since mitochondrial function is tightly linked to mitochondrial dynamics, the expression of key genes regulating mitochondrial fusion and fission was further analyzed. No significant differences were observed between the OP and GelMA groups. In contrast, Mn‐ZIF‐8@GelMA treatment significantly downregulated the mitochondrial fission‐related genes *dnm1l* and *fis1*, while upregulating the fusion‐related genes *mfn1*, *mfn2*, and *opa1* (Figure 5D), suggesting a shift toward a more balanced mitochondrial dynamic state.

Among the tested formulations, M‐Gel (0.3% Mn‐ZIF‐8@GelMA) exhibited the most pronounced regulatory effects on osteogenic differentiation and mitochondrial function. We further confirmed its effects on the early osteogenic and migratory capacities of OP‐mBMSCs (Figure S9). Therefore, M‐Gel was selected as the representative Mn‐ZIF‐8@GelMA formulation for subsequent analyses.

We next examined whether these functional improvements were accompanied by changes in mitochondrial morphology. Mitochondria in the OP and GelMA groups appeared predominantly short and spherical, whereas mitochondria in the Mn‐ZIF‐8@GelMA group were elongated and formed interconnected network‐like structures (Figure 5E). Quantitative analysis demonstrated significant increases in mitochondrial length, branching, and distribution area after Mn‐ZIF‐8@GelMA treatment, indicating improvement of mitochondrial morphology and recovery of mitochondrial network integrity.

As described above, intracellular ROS accumulation and mitochondrial dysfunction were simultaneously observed in OP‐mBMSCs, while Mn‐ZIF‐8@GelMA treatment concurrently reduced intracellular ROS levels and improved mitochondrial function. To further investigate the relationship between ROS accumulation and mitochondrial dysfunction, we further treated OP‐mBMSCs with the mitochondria‐targeted antioxidant (MitoTEMPO) and the nicotinamide adenine dinucleotide phosphate oxidase (NOX) inhibitor (VAS2870) to determine the contribution of mitochondrial‐derived and non‐mitochondrial ROS to the oxidative stress state (Figure S10). The results showed that both VAS2870 and MitoTEMPO treatment reduced intracellular ROS levels and mitochondrial ROS accumulation in OP‐mBMSCs, suggesting that both non‐mitochondrial and mitochondrial ROS sources contribute to the oxidative stress state under osteoporotic conditions. Notably, MitoTEMPO treatment produced a more pronounced reduction in intracellular ROS levels, indicating that mitochondrial‐derived ROS represents an important contributor to the overall oxidative burden. In contrast, Mn‐ZIF‐8@GelMA simultaneously reduced intracellular and mitochondrial ROS accumulation, suggesting its antioxidant effect involves the regulation of multiple ROS sources rather than targeting a single pathway.

Together, these findings suggest that oxidative stress and mitochondrial abnormalities represent interconnected features of OP‐mBMSC dysfunction. Although ZIF‐8@GelMA exhibited certain effects in improving osteogenic differentiation and reducing oxidative stress in OP‐mBMSCs, Mn‐ZIF‐8@GelMA showed more pronounced ROS‐scavenging capacity and stronger pro‐osteogenic effects, suggesting that Mn incorporation provides additional redox‐regulatory activity beyond the intrinsic biological effects of ZIF‐8/ Zn^2+^ (Figure S12). Mn‐ZIF‐8@GelMA mitigates excessive ROS accumulation from both mitochondrial and non‐mitochondrial sources while improving mitochondrial functional and structural characteristics, which may contribute to the enhancement of osteogenic capacity under osteoporotic conditions. Nevertheless, the precise contributions of Mn and Zn ions cannot be fully distinguished based on the current experimental design. The use of free Mn^2+^ as a direct control was limited by its reduced cellular compatibility, likely due to differences in ion availability and release behavior compared with Mn incorporated within the ZIF‐8 framework (Figure S11). Future studies using more refined ion‐specific control systems will be needed to further clarify the independent biological roles of Mn and Zn.

### 2.4 Potential Mechanism Underlying Mn‐ZIF‐8@GelMA Activity

#### 2.4.1 RNA Sequencing Analysis

To further explore the molecular basis by which Mn‐ZIF‐8@GelMA rescued the osteogenic potential and mitochondrial function of OP‐mBMSCs, RNA sequencing was performed to characterize the transcriptomic changes induced by hydrogel treatment. Heatmap visualization showed a clear separation between OP‐mBMSCs in the GelMA group and those treated with Mn‐ZIF‐8@GelMA, indicating that the composite hydrogel induced a broad transcriptional response rather than merely causing minor stochastic gene fluctuations (Figure 6A). A total of 6107 differentially expressed genes (DEGs) were identified, including 3054 upregulated and 3053 downregulated genes (Figure 6B). The RT‐qPCR results of several representative genes were consistent with the sequencing data, confirming the reliability of the transcriptomic analysis (Figure 6C).

**FIGURE 6:**
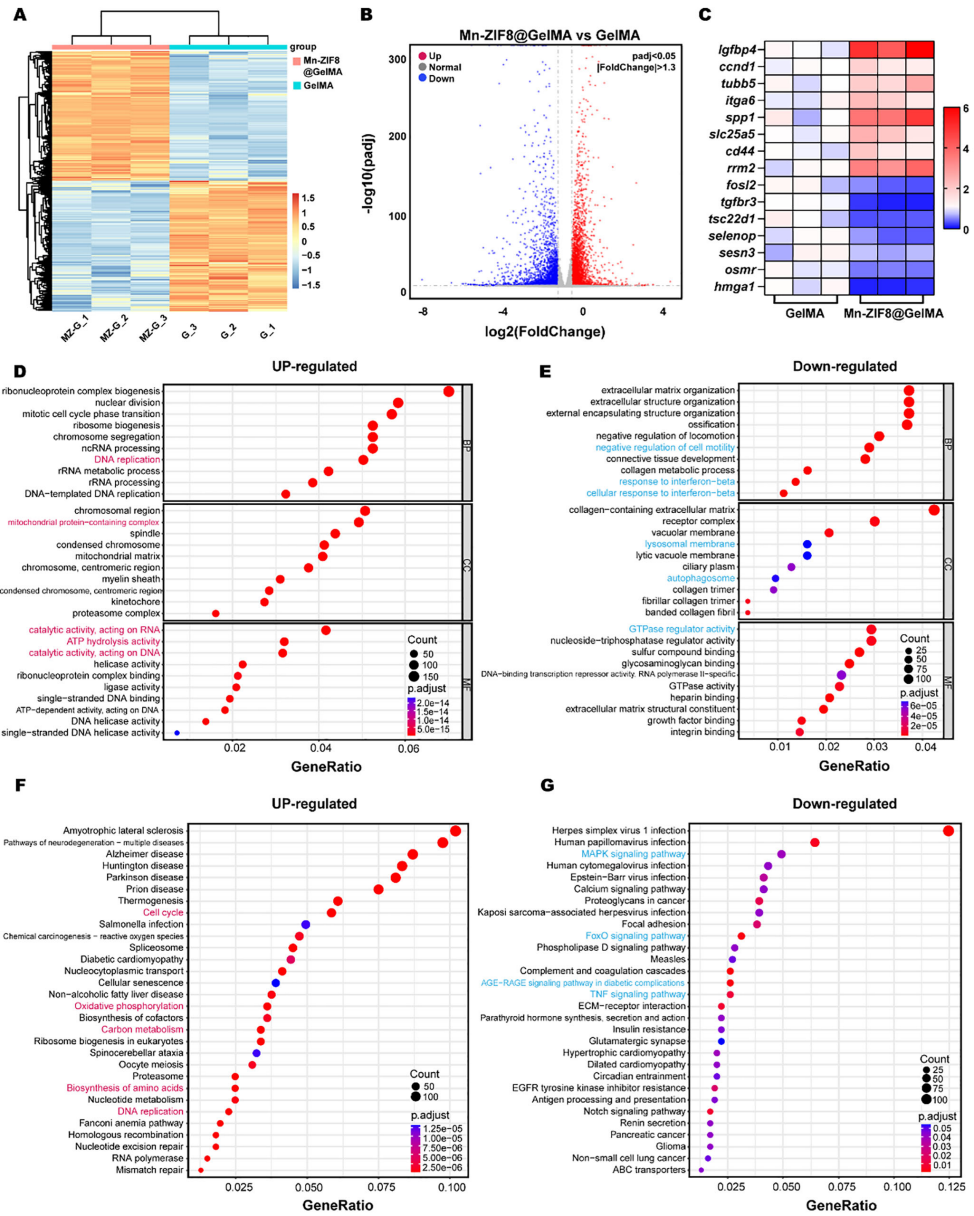
Transcriptomic analysis of OP‐mBMSCs treated with Mn‐ZIF‐8@GelMA hydrogels. (A) Heatmap showing hierarchical clustering of differentially expressed genes (DEGs) between GelMA and Mn‐ZIF‐8@GelMA groups (n = 3 each group). (B) Volcano plot illustrating DEGs, with red dots indicating upregulated genes and blue dots indicating downregulated genes (|Fold Change|≥1.3, *p* < 0.05). (C) Heatmap of representative DEGs associated with osteogenesis, mitochondrial function, and inflammation. (D) GO enrichment analysis of upregulated DEGs. (E) GO enrichment analysis of downregulated DEGs. (F) KEGG pathway enrichment of upregulated DEGs. (G) KEGG pathway enrichment of downregulated DEGs.

Gene Ontology (GO) enrichment analysis was performed to further characterize the biological processes and functional modules affected by Mn‐ZIF‐8@GelMA treatment (Figure 6D,E). The upregulated genes were predominantly enriched in terms related to DNA replication, mitochondrial protein‐containing complexes, catalytic activity, and ATP hydrolysis, suggesting that Mn‐ZIF‐8@GelMA enhanced biosynthetic activity, mitochondrial complex‐associated functions, and energy‐dependent cellular processes in OP‐mBMSCs. This transcriptional pattern is consistent with the observed improvement of mitochondrial membrane potential and ATP production, supporting the notion that Mn‐ZIF‐8@GelMA does not merely reduce oxidative burden but may actively recover mitochondrial bioenergetic competence. Previous studies have shown that mitochondrial oxidative phosphorylation is increased during osteogenic commitment of MSCs, and inhibition of mitochondrial respiration can impair osteogenic differentiation, highlighting the close relationship between mitochondrial metabolism and bone‐forming potential [53, 54].

In contrast, downregulated genes were enriched in terms associated with negative regulation of cell motility, interferon‐β response, lysosomal membrane, autophagosome formation, and GTPase regulatory activity. These changes suggest that Mn‐ZIF‐8@GelMA attenuated stress‐related transcriptional programs while relieving inhibitory signals that may restrict cellular migration and functional recovery. Autophagy is a context‐dependent process that may either protect cells by removing damaged organelles or reflect an increased burden of cellular stress [55]. In the present setting, together with the reduced ROS level, improved mitochondrial morphology, and increased ATP content, the decreased enrichment of autophagy‐related terms may indicate a lower requirement for stress‐induced organelle clearance rather than a simple suppression of basal homeostatic autophagy. This interpretation is supported by previous studies showing that mitophagy is often activated as a protective response to oxidative stress and mitochondrial damage in bone‐related cells and MSCs [56].

Kyoto Encyclopedia of Genes and Genomes (KEGG) pathway enrichment analysis further supported this mitochondrial‐centered transcriptional remodeling (Figure 6F,G). Upregulated pathways included cell cycle, oxidative phosphorylation, carbon metabolism, amino acid biosynthesis, and DNA replication, indicating that Mn‐ZIF‐8@GelMA promoted a transcriptional state characterized by enhanced mitochondrial bioenergetics, anabolic metabolism, and proliferative capacity [57, 58]. In contrast, the downregulated KEGG pathways were mainly related to stress‐responsive and inflammatory signaling, which are closely associated with the pathological microenvironment of osteoporosis [59].

Excessive ROS can act as upstream signaling molecules that activate mitogen‐activated protein kinase (MAPK) cascades, including extracellular regulated protein kinases (ERK), c‐Jun N‐terminal kinase (JNK), and p38, thereby amplifying stress‐responsive transcriptional programs in bone‐related cells [60]. Pro‐inflammatory cytokines such as TNF‐α, IL‐1β, and IL‐6 are also closely associated with inflammatory bone loss, partly by enhancing RANKL‐dependent osteoclast differentiation and bone‐resorptive activity [61]. TNF‐related inflammatory signaling can further disrupt bone remodeling by promoting osteoclastogenesis while impairing osteoblast differentiation and matrix‐forming capacity [62]. Therefore, the downregulation of MAPK and TNF signaling pathways in Mn‐ZIF‐8@GelMA‐treated OP‐mBMSCs may indicate attenuation of excessive inflammatory and oxidative stress signaling rather than nonspecific suppression of cell activity.

Forkhead box O (FoxO) signaling represents another stress‐adaptive pathway closely linked to cellular redox homeostasis, because FoxO transcription factors are activated under oxidative stress and participate in antioxidant defense programs in osteoblast‐lineage cells [63, 64]. However, sustained FoxO activation may antagonize osteogenic signaling by diverting β‐catenin away from T‐cell factor mediated Wnt transcription toward FoxO‐dependent stress‐response transcription [65]. Thus, the reduced enrichment of FoxO signaling after Mn‐ZIF‐8@GelMA treatment may reflect relief from a chronic oxidative stress‐adaptive state, allowing OP‐mBMSCs to regain a transcriptional environment more favorable for osteogenic differentiation. AGE‐RAGE signaling has been implicated in impaired bone remodeling, as activation of RAGE signaling has been reported to inhibit osteogenic differentiation and facilitate osteoclastogenesis in OP‐related settings [66]. The downregulation of the AGE‐RAGE pathway may indicate attenuation of damage‐associated metabolic and inflammatory stress. Together with the reduced enrichment of MAPK, FoxO, and TNF signaling, this finding supports the view that Mn‐ZIF‐8@GelMA shifts OP‐mBMSCs away from a maladaptive stress‐dominated state and toward a transcriptional environment more permissive for functional recovery.

Collectively, these results suggest that Mn‐ZIF‐8@GelMA promotes a transcriptional shift toward enhanced mitochondrial metabolism and reduced cellular stress, which may contribute to the improvement of BMSC function under osteoporotic conditions.

#### 2.4.2 Mn‐ZIF‐8@GelMA Rescues Mitochondrial Dynamics Through MAVS‐mediated Suppression of DRP1‐dependent Fission

Based on the transcriptomic analysis, we next sought to identify the mitochondrial regulatory signals that may link Mn‐ZIF‐8@GelMA‐induced transcriptional remodeling to the improvement of OP‐mBMSC function. Functional enrichment analysis revealed that these DEGs were significantly associated with mitochondrial‐related biological processes, including mitochondrial protein complexes, oxidative phosphorylation, and ATP‐associated metabolic processes. These transcriptomic changes were consistent with our previous observations that Mn‐ZIF‐8@GelMA improved mitochondrial membrane potential, increased ATP production, and promoted mitochondrial network organization, suggesting that mitochondrial regulation represents an important component of the biological response induced by Mn‐ZIF‐8@GelMA.

Genes associated with mitochondrial localization or mitochondrial functional regulation were selected based on their annotated cellular localization and biological functions. Considering that OP‐mBMSCs exhibited excessive DRP1‐dependent mitochondrial fission characterized by increased DRP1 Ser616 phosphorylation and impaired mitochondrial morphology, we focused on genes potentially involved in mitochondrial signaling and dynamics regulation. Among these candidates, *mavs*, encoding mitochondrial antiviral signaling protein (MAVS), emerged as a potential candidate.

MAVS is a mitochondrial outer membrane‐localized adaptor protein originally identified as a central mediator of antiviral innate immune signaling [67]. Beyond its canonical role in antiviral responses, MAVS has increasingly been recognized as a mitochondrial signaling molecule involved in the regulation of mitochondrial homeostasis, cellular stress responses, and metabolic adaptation [68]. Rather than interpreting MAVS solely as a classical antiviral molecule, we considered it a potential mitochondrial signaling node that may connect Mn‐ZIF‐8@GelMA treatment with the recovery of mitochondrial homeostasis in OP‐mBMSCs.

Previous studies have shown that oxidative stress and inflammatory stimulation can induce excessive DRP1‐dependent mitochondrial fission, and that DRP1‐mediated fission contributes to mitochondrial dysfunction and impaired osteogenic differentiation in osteoblasts [17, 18]. Consistent with these findings, our earlier morphological observations showed that mitochondria in OP‐mBMSCs were shortened, swollen, and more spherical, suggesting disruption of the mitochondrial network. At the molecular level, both total DRP1 and phosphorylated DRP1 at Ser616 were markedly increased in OP‐mBMSCs, indicating enhanced mitochondrial fission activity. Phosphorylation of DRP1 at Ser616 is widely associated with increased mitochondrial fission activity through enhanced DRP1 recruitment and activation [69]. In parallel, the expression of MAVS and the osteogenic marker BMP‐2 was reduced under osteoporotic conditions (Figure S13), suggesting that OP‐mBMSCs were characterized by MAVS deficiency, excessive DRP1‐associated mitochondrial fission, and impaired osteogenic potential.

We then investigated whether Mn‐ZIF‐8@GelMA could reverse this abnormal mitochondrial dynamic state. Compared with the OP and GelMA groups, Mn‐ZIF‐8@GelMA markedly rescued MAVS expression at both the transcriptional and protein levels (Figure 7A,B). This change was accompanied by a significant reduction in DRP1 Ser616 phosphorylation, indicating that the excessive activation of DRP1‐dependent mitochondrial fission was attenuated after Mn‐ZIF‐8@GelMA treatment. Meanwhile, BMP‐2 expression was substantially increased in the Mn‐ZIF‐8@GelMA group, linking the improvement of mitochondrial dynamic balance with enhanced osteogenic potential. Together, these results suggest that Mn‐ZIF‐8@GelMA shifts OP‐mBMSCs from a mitochondria‐fragmented, osteogenesis‐impaired state toward a more functionally competent phenotype characterized by rescued MAVS expression, restrained DRP1 activation, and improved osteogenic marker expression.

**FIGURE 7:**
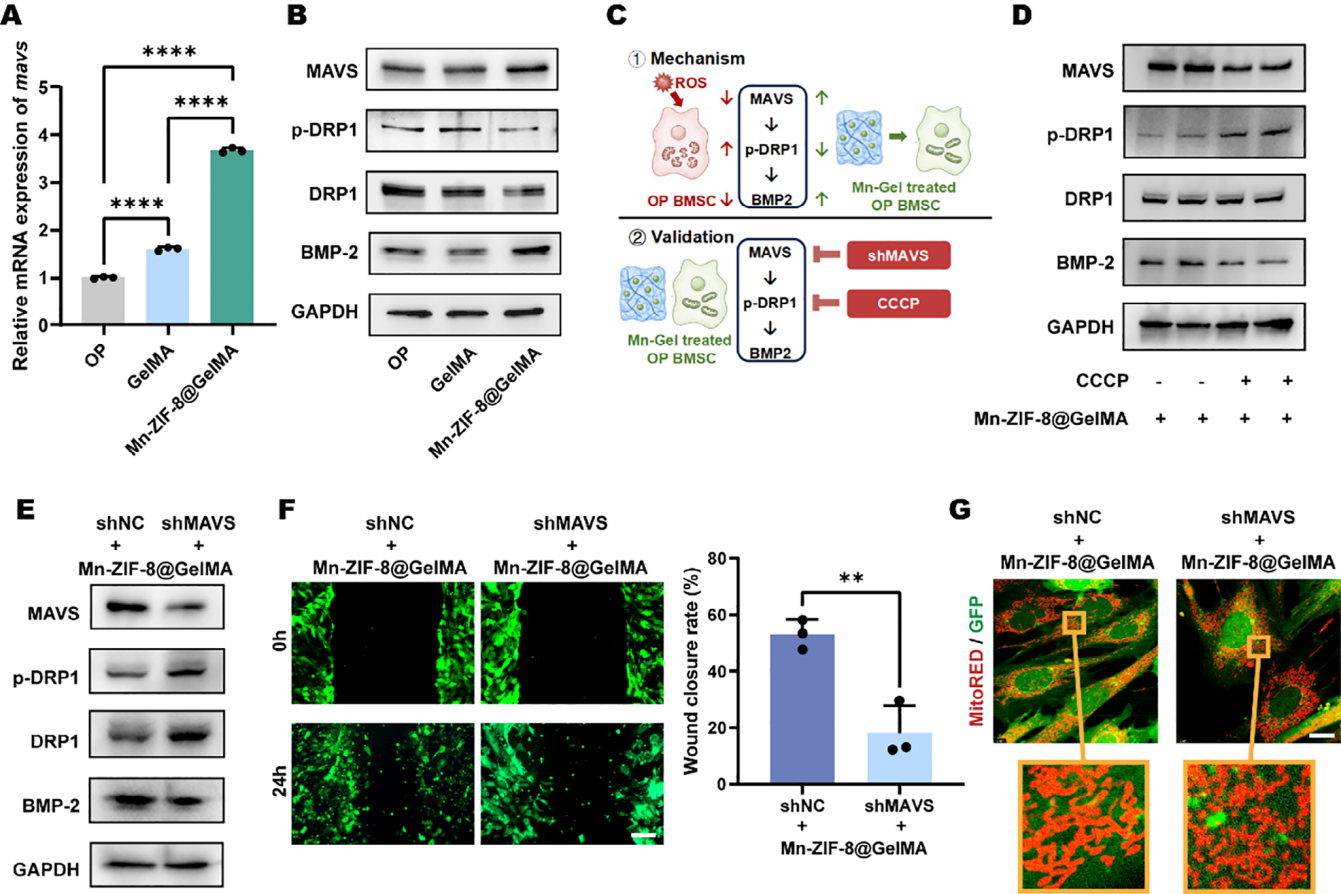
MAVS mediates the regulatory effect of Mn‐ZIF‐8@GelMA in OP‐mBMSCs. (A) Relative mRNA expression of *mavs* in different groups (*n* = 3 each group). (B) Western blot analysis showing the expression of MAVS, phosphorylated DRP1 (p‐DRP1), DRP1, and BMP‐2 in each group (*n* = 3 each group). GAPDH was used as a loading control. (C) Schematic diagram illustrating the proposed mechanism. (D) Western blot analysis showing the effect of mitochondrial uncoupler CCCP on MAVS, p‐DRP1, DRP1, and BMP‐2 expression in Mn‐ZIF‐8@GelMA‐treated cells (*n* = 3 each group). (E) Western blot analysis of MAVS, p‐DRP1, DRP1, and BMP‐2 in OP‐mBMSCs transfected with shNC or shMAVS (*n* = 3 each group). (F) Representative fluorescence images of cell migration and quantitative analysis of wound closure in Mn‐ZIF‐8@GelMA‐treated OP‐mBMSCs transfected with shNC or shMAVS at 0 and 24 h (*n* = 3 each group). Scale bar: 500 µm. (G) Confocal microscopy images of mitochondrial morphology in shNC and shMAVS groups (*n* = 3 each group). Enlarged images demonstrate that MAVS knockdown disrupts mitochondrial network integrity. Error bars indicate the mean ± SD. Scale bar: 20 µm. ^*^ *p* < 0.05, ^**^ *p* < 0.01, ^***^ *p* < 0.001, ^****^ *p* < 0.0001.

To further validate the mechanism, we conducted blocking experiments targeting both the mitochondria function and MAVS signaling pathways (Figure 7C). First, Mn‐ZIF‐8@GelMA‐treated OP‐mBMSCs were exposed to carbonyl cyanide m‐chlorophenyl hydrazone (CCCP), a mitochondrial uncoupler which can induce mitochondrial stress and trigger mitochondrial fragmentation [70]. CCCP treatment markedly increased p‐DRP1 Ser616 levels and partially reversed the Mn‐ZIF‐8@GelMA‐induced upregulation of MAVS and BMP‐2 (Figure 7D). These results indicate that disruption of mitochondrial homeostasis compromises the ability of Mn‐ZIF‐8@GelMA to restrain excessive mitochondrial fission and rescue osteogenic signaling in OP‐mBMSCs. The pro‐osteogenic effect of Mn‐ZIF‐8@GelMA is closely linked to its capacity to maintain mitochondrial homeostasis and regulate the MAVS/DRP1 axis.

On the other hand, to determine whether MAVS was functionally involved in this mitochondrial regulatory process, MAVS expression was knocked down in OP‐mBMSCs using lentiviral shRNA, with shNC serving as the control. Both groups were then treated with Mn‐ZIF‐8@GelMA. Loss of MAVS markedly increased p‐DRP1 while reducing BMP‐2 expression (Figure 7E), which supports a MAVS‐DRP1‐associated mechanism in the regulation of osteogenic competence. Functionally, MAVS knockdown impaired cell migration capacity and induced a fragmented and punctate mitochondrial phenotype with loss of network integrity (Figure 7F,G). These results showed that MAVS is partially involved in maintaining mitochondrial dynamic balance and supporting the functional recovery of OP‐mBMSCs.

Taken together, these results indicate that MAVS participates in the regulation of mitochondrial dynamic balance in OP‐mBMSCs. Specifically, Mn‐ZIF‐8@GelMA suppresses DRP1‐driven excessive mitochondrial fission, preserves mitochondrial network integrity, and ultimately rescues the osteogenic competence of osteoporotic BMSCs.

### 2.5 Mn‐ZIF‐8@GelMA Accelerates OP Extraction Socket Repair In Vivo

Based on the pro‐osteogenic and mitochondrial function‐restoring effects observed in vitro, the 0.3% Mn‐ZIF‐8‐loaded GelMA hydrogel was further evaluated in an osteoporotic maxillary first molar extraction model to determine its biosafety and regenerative efficacy in vivo (Figure S14). The systemic biosafety of the composite hydrogel was first assessed by histological examination of major organs. H&E staining of the heart, liver, spleen, lung, and kidney showed preserved tissue architecture with no obvious inflammatory infiltration, tissue necrosis, or structural abnormalities (Figure S15), indicating that local implantation of Mn‐ZIF‐8@GelMA did not cause detectable histopathological injury in major organs during the observation period.

Micro‐CT analysis revealed limited mineralized tissue formation and incomplete socket repair in the Blank group (Figure 8A,B). This observation is consistent with the pathological features of OP, in which impaired bone formation, excessive bone resorption, and dysfunctional osteoprogenitor activity collectively delay bone repair [71]. GelMA treatment partially improved socket filling, likely owing to its injectable ECM‐like scaffold structure that provides temporary spatial support for tissue ingrowth [72]. Mn‐ZIF‐8@GelMA produced the most pronounced regenerative effect, as evidenced by greater mineralized bone formation, denser trabecular‐like architecture, and improved defect filling compared with both the Blank and GelMA groups. These findings indicate that incorporation of Mn‐ZIF‐8 transformed GelMA from a passive structural scaffold into a bioactive regenerative platform capable of enhancing osteoporotic socket bone repair.

**FIGURE 8:**
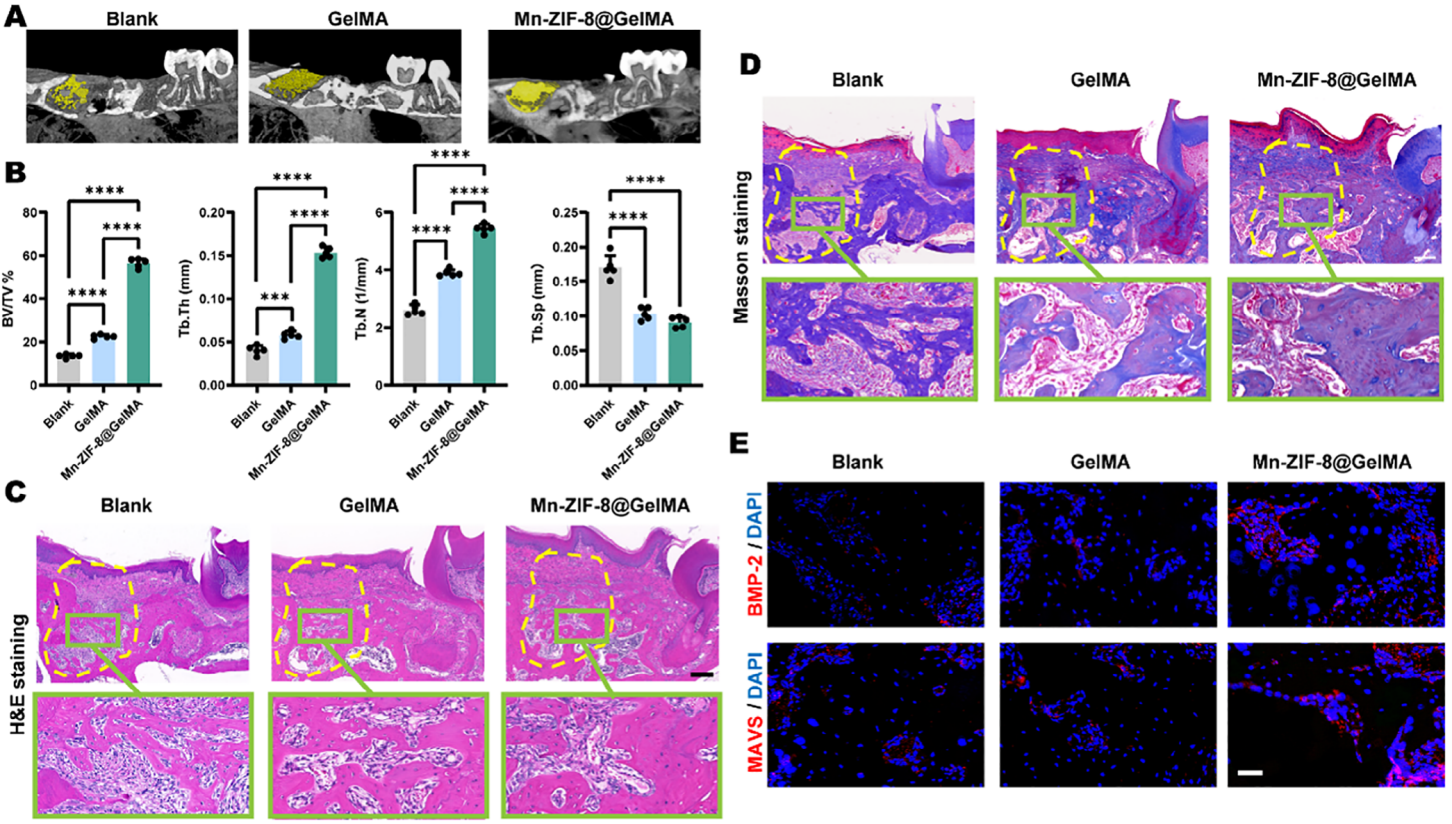
Mn‐ZIF‐8@GelMA promotes alveolar bone regeneration in vivo. (A) Representative micro‐CT images of alveolar bone healing in Blank, GelMA, and Mn‐ZIF‐8@GelMA groups at 14 days post‐extraction (*n* = 5 each group). Yellow regions indicate the ROI used for quantitative analysis. (B) Quantitative analysis of bone microarchitecture parameters, including bone volume fraction (BV/TV), trabecular thickness (Tb.Th), trabecular number (Tb.N) and trabecular separation (Tb.Sp) (*n* = 5 each group). (C) H&E staining showing new bone formation and tissue organization at the extraction sites of different groups. Enlarged images highlight newly formed trabeculae (*n* = 5 each group). Scale bar: 200 µm. (D) Masson's trichrome staining demonstrating collagen deposition and bone matrix maturation in each group (*n* = 5 each group). Enlarged regions show enhanced collagen fiber formation. Scale bar: 200 µm. (E) Immunofluorescence staining of BMP‐2 and MAVS expression in regenerated alveolar bone tissue, with nuclei counterstained by DAPI. Error bars indicate the mean ± SD. Scale bar: 40 µm. ^*^ *p* < 0.05, ^**^ *p* < 0.01, ^***^ *p* < 0.001, ^****^ *p* < 0.0001.

Current knowledge of extraction socket healing indicates that clot organization, connective tissue formation, woven bone deposition, and subsequent remodeling occur as coordinated stages rather than isolated events [73]. Histological analysis further confirmed the superior tissue repair induced by Mn‐ZIF‐8@GelMA. H&E staining showed sparse newly formed bone and incomplete defect filling in the Blank group, whereas GelMA promoted partial bone formation but left residual voids within the socket region. In contrast, Mn‐ZIF‐8@GelMA resulted in more extensive and better‐organized trabecular bone formation, suggesting accelerated structural reconstruction of the extraction socket (Figure 8C). Masson's trichrome staining showed a similar trend in extracellular matrix remodeling. Mn‐ZIF‐8@GelMA induced abundant and more organized collagen fiber deposition, indicating improved matrix maturation and wound remodeling (Figure 8D). Together, these results suggest that the composite hydrogel promoted not only mineralized bone formation but also extracellular matrix organization, both of which are essential for effective socket repair. Notably, no obvious residual hydrogel structures were observed within the extraction sockets at day 14, suggesting that Mn‐ZIF‐8@GelMA provided a transient regenerative microenvironment during the early healing phase rather than acting as a long‐term physical scaffold. The precise in vivo degradation kinetics and residual amount of the hydrogel were not quantitatively evaluated in the present study. Future studies incorporating longitudinal imaging or histological tracing approaches will further elucidate the dynamic relationship between hydrogel degradation and bone regeneration.

Consistent with the in vitro findings, Mn‐ZIF‐8@GelMA also enhanced osteogenic and mitochondrial regulatory signals in vivo. Immunofluorescence staining showed stronger BMP‐2 expression in the regenerated socket tissue, indicating increased osteogenic activity [74]. Importantly, MAVS expression was also elevated in the Mn‐ZIF‐8@GelMA groups (Figure 8E). This in vivo expression pattern supports the proposed link between mitochondrial regulatory signaling and osteogenic regeneration, suggesting that the regenerative effect of Mn‐ZIF‐8@GelMA is associated with improvement of an osteogenesis‐supportive mitochondrial state.

Collectively, Mn‐ZIF‐8@GelMA effectively accelerated extraction socket repair under osteoporotic conditions, as demonstrated by improved mineralized bone formation, enhanced trabecular organization, increased collagen matrix maturation, and elevated BMP‐2 expression. The concomitant increase in MAVS further supports the involvement of mitochondrial homeostasis in this regenerative process. These findings indicate that Mn‐ZIF‐8@GelMA functions not merely as an injectable socket‐filling scaffold, but as a bioactive microenvironment‐modulating hydrogel that promotes osteoporotic alveolar bone regeneration.

## 3 Conclusion

In summary, this study proposes a mitochondrial homeostasis‐oriented biomaterial strategy for enhancing osteoporotic jawbone defect regeneration. We developed a redox‐active Mn/Zn bimetallic nanoparticle‐loaded GelMA hydrogel to modulate the pathological microenvironment of osteoporotic bone. By reducing excessive ROS accumulation and restoring mitochondrial functional homeostasis, the composite hydrogel rescued the impaired osteogenic capacity of OP‐mBMSCs and significantly promoted new bone formation within osteoporotic extraction sockets.

Mechanistically, our results reveal that Mn‐ZIF‐8@GelMA rescues mitochondrial dynamic balance through activation of MAVS and suppression of DRP1‐mediated mitochondrial fission, thereby alleviating oxidative stress‐induced mitochondrial dysfunction and re‐establishing cellular bioenergetic capacity. This MAVS‐DRP1‐mitochondrial dynamics regulatory axis provides a mechanistic explanation for how mitochondrial homeostasis governs the osteogenic potential of BMSCs under osteoporotic conditions.

These findings highlight mitochondrial dysfunction as a central therapeutic target for osteoporotic bone regeneration and establish a clinically translatable approach in which locally delivered biomaterials rescue stem cell bioenergetic homeostasis to overcome regeneration resistance. This work therefore provides both mechanistic insight into osteoporotic jawbone defect repair and a promising strategy for the development of multifunctional biomaterials for regenerative dentistry.

## 4 Materials and Methods

### 4.1 Osteoporotic Animal Model

All animal experiments were approved by the Animal Ethics Committee of the Ninth People's Hospital, Shanghai Jiao Tong University School of Medicine (Approval No. SH9H‐2023‐A890‐1) and conducted in accordance with the institutional guidelines for the care and use of laboratory animals. The grouping and modeling procedures are illustrated schematically in the supplementary Figure S1A. Eight‐week‐old female C57BL/6 mice were housed in a specific pathogen‐free (SPF) environment with free access to food and water. The mice were randomly divided into two groups. Bilateral ovariectomy (OVX) was performed to induce osteoporosis, whereas mice in the control group underwent sham surgery, in which only a portion of the adipose tissue surrounding the ovaries was removed. Eight weeks after surgery, the establishment of the osteoporotic model was verified by micro‐computed tomography (micro‐CT) analysis of the femur.

### 4.2 Isolation, Culture, and Identification of Primary mBMSCs

Primary mBMSCs were isolated using the whole bone marrow adherence method. Bone marrow was harvested from the femurs and tibias of mice in the Ctrl and OP groups and cultured in α‐minimum essential medium (α‐MEM) (Hyclone, USA) supplemented with 10% fetal bovine serum (Beyotime, China) and 1% penicillin‐streptomycin (Gibco, USA). Cells were maintained in a humidified incubator at 37°C with 5% CO_2_ and 95% humidity. When adherent cells reached approximately 90% confluence without obvious floating cells, they were passaged using 0.25% EDTA‐trypsin (Gibco, USA). Cells at passages P2‐P3 were seeded at appropriate densities for subsequent experiments. The multipotent differentiation capacity of the isolated cells was confirmed by osteogenic and adipogenic induction followed by Alizarin Red S (ARS) staining and Oil Red O staining, respectively.

### 4.3 Synthesis of Mn‐ZIF‐8 Nanoparticles and Composite Hydrogel

For the synthesis of ZIF‐8 nanoparticles, a zinc salt solution (Zn(AC)_2_·2H_2_O, Sigma‐Aldrich, USA; 1 mM) was slowly added to a 2‐methylimidazole solution (2‐MI, Sigma–Aldrich, USA; 60 mM) at room temperature, followed by magnetic stirring at 600 rpm for 10 s. After standing for 6 h, ZIF‐8 nanoparticles were formed. The precipitate was collected by centrifugation at 7000 rpm, washed three times with anhydrous ethanol, and dried overnight in an oven at 70°C.

For the preparation of Mn‐ZIF‐8, Mn(AC)_2_·4H_2_O (Sigma–Aldrich, USA) was added to the zinc salt solution to achieve a Mn/(Mn+Zn) molar ratio of 20%, while the remaining preparation steps were identical to those used for ZIF‐8.

To fabricate the composite hydrogel, a 10% (w/v) GelMA solution was prepared and supplemented with 0.25% (w/v) photoinitiator LAP (Engineering for Life, China). The mixture was heated in a 70°C water bath for 30 min under light‐protected conditions until fully dissolved. Mn‐ZIF‐8 nanoparticles were then added to the GelMA solution at different concentrations and dispersed by ultrasonication. The precursor solution was injected into molds and exposed to 405 nm ultraviolet light for 60 s to form the composite hydrogel.

### 4.4 Material Characterization

The surface morphology of nanoparticles was observed using scanning electron microscopy (SEM) (ZEISS Sigma 300, Germany). Transmission electron microscopy (TEM) (JEOL JEM‐F200, Japan) was used to examine the crystal morphology and internal structure of the nanoparticles, and elemental mapping was performed to evaluate the distribution of Mn, Zn, and other elements. For Mn‐ZIF‐8@GelMA hydrogels, samples were freeze‐dried and their cross‐sections were sputter‐coated with gold prior to SEM observation to observe the pore structure. Elemental mapping was also conducted to determine the elemental composition within the hydrogel network.

X‐ray photoelectron spectroscopy (XPS) (Thermo Scientific K‐Alpha, USA) was employed to analyze the chemical valence states of Mn in Mn‐ZIF‐8 and to confirm the coexistence of multiple oxidation states that may contribute to redox regulation. X‐ray diffraction (XRD) (Rigaku SmartLab SE, Japan) was further performed to analyze the crystal structure and chemical composition of Mn‐ZIF‐8 and Mn‐ZIF‐8@GelMA. The mechanical properties of the hydrogels were evaluated using a universal testing machine (Instron, USA). Cylindrical hydrogel samples with a diameter of 20 mm were prepared and subjected to tensile and compressive testing at room temperature with a loading rate of 1 mm/min. The tensile and compressive moduli were calculated from the slope of the linear region within 0%–10% strain.

To evaluate the degradation behavior of nanoparticles within the composite hydrogel, hydrogels were immersed in a defined volume of phosphate‐buffered saline (PBS) and incubated at 37°C. At predetermined time points (12 h, 1 day, 3 days, 5 days, 7 days, 14 days, and 21 days), the concentration of released Mn ions in the buffer solution was measured using inductively coupled plasma mass spectrometry (ICP‐MS). Ion release curves were subsequently generated.

To determine the optimal nanoparticle concentration within the composite hydrogel, cell proliferation was assessed using a CCK‐8 assay. During the preliminary screening, hydrogels with different concentrations were co‐cultured with cells for 24 h. Based on the results, further screening experiments were conducted with a 72 h co‐culture period. For the assay, the original culture medium was removed and replaced with fresh medium containing 10% CCK‐8 reagent (Beyotime, China). After incubation at 37°C for 1 h, the absorbance at 450 nm was measured and the optical density (OD) values were recorded.

To evaluate the hemostatic performance of Mn‐ZIF‐8@GelMA, a mouse liver injury model was established. Seven‐week‐old C57BL/6 mice weighing 18–20 g were anesthetized as previously described, and the liver was surgically exposed. A linear incision approximately 5 mm in length was created on the liver surface using surgical scissors. After spontaneous bleeding for 2 s, an appropriate amount of hydrogel precursor was applied onto the wound site and subsequently photocrosslinked in situ. A pre‐weighed filter paper was placed beneath the incision site to collect blood loss. After complete hemostasis was achieved, the filter paper was immediately collected and weighed using an electronic balance to calculate the blood loss.

### 4.5 Antibacterial Activity

*Streptococcus mutans* (*S. mutans*, ATCC 25175) was cultured in brain heart infusion (BHI) broth, and *Escherichia coli (E. coli*, ATCC 25922) was cultured in Luria‐Bertani (LB) medium at 37°C for 48 h and subcultured for subsequent experiments. Sterilized hydrogel samples were placed in 24‐well plates before the addition of bacterial suspensions. For bacterial growth assessment, the bacterial suspensions were adjusted to 1 × 10^4^ CFU/mL and incubated with the hydrogel samples at 37°C for 24 h. After incubation, bacterial growth was evaluated by measuring the optical density at 600 nm using a microplate reader (Tecan, Switzerland).

Bacterial viability was further evaluated using a LIVE/DEAD BacLight bacterial viability kit (Thermo Fisher Scientific, USA). Bacterial suspensions were adjusted to 1 × 10^6^ CFU/mL and incubated with the hydrogel samples at 37°C for 24 h. After incubation with the hydrogel samples, bacteria were collected and stained with SYTO 9 and propidium iodide (PI) according to the manufacturer's instructions and observed under a fluorescence microscope. Images were acquired using a fluorescence microscope. Live bacteria exhibited green fluorescence, whereas dead bacteria exhibited red fluorescence. The red and green fluorescence signals were quantified using ImageJ software, and the proportion of live and dead bacteria was qualitatively evaluated to assess antibacterial performance.

### 4.6 Scratch Wound Assay

Primary mBMSCs were subjected to the corresponding experimental treatments before the assay. After treatment, cells in good condition were counted and seeded into six‐well plates at a density of 5 × 10^5^ cells per well. When the cells reached approximately 95% confluence, a linear scratch was generated across the cell monolayer using a sterile 200 µL pipette tip. The wells were washed three times with PBS to remove detached cells, and the culture medium was subsequently replaced with serum‐free medium to minimize the influence of cell proliferation. Images were captured using an inverted microscope immediately after scratching and at the indicated time points. The residual wound area was quantified using ImageJ software, and the wound closure percentage was calculated as follows: Wound closure (%) = (A_0_‐A_t_) /A_0_*100, where A_0_ represents the initial wound area at 0 h and A_t_ represents the residual wound area at the indicated time point.

### 4.7 In Vitro Antioxidant Activity Evaluation

The SOD‐like activity was assessed using a WST‐8 total superoxide dismutase assay kit (Beyotime, China). Superoxide anions (•O_2_^−^) are generated through the enzymatic reaction catalyzed by xanthine oxidase. Materials with SOD‐like activity facilitate the elimination of •O_2_^−^, thereby reducing its reaction with WST‐8 and decreasing the formation of water‐soluble colored products. After completion of the reaction, the absorbance of each group was measured at 450 nm, and the •O_2_^−^ scavenging rate was calculated.

The CAT‐like activity was evaluated using a catalase activity assay kit (Beyotime, China). Different materials were incubated with H_2_O_2_, and the residual H_2_O_2_ in the reaction system was subsequently detected through a peroxidase‐mediated colorimetric reaction. The remaining H_2_O_2_ oxidizes the chromogenic substrate under peroxidase catalysis, generating a red quinoneimine dye product. After the reaction, the absorbance was measured at 520 nm. The residual H_2_O_2_ concentration was determined based on the standard curve and calculation method provided by the manufacturer, and the H_2_O_2_ scavenging rate was subsequently calculated.

The hydroxyl radical (•OH) scavenging capability was evaluated using a Fenton reaction‐based assay. Hydroxyl radicals were generated through the reaction between FeSO_4_ and H_2_O_2_ (Fe^2+^ + H_2_O_2_ → Fe^3+^ + •OH + OH‐). The generated •OH further oxidized TMB to produce a blue‐colored oxidation product (oxTMB). The •OH scavenging capability was evaluated by assessing the inhibition of TMB oxidation. After completion of the reaction, UV–vis absorption spectra ranging from 500 to 800 nm were recorded to characterize the formation of oxTMB.

### 4.8 Mitochondrial Function Associated Assessment

Intracellular ROS levels were detected using the fluorescent probe 2’,7’‐dichlorofluorescein diacetate (DCFH‐DA) (Beyotime, China). Mitochondrial ROS levels were detected using the Mitochondrial Superoxide Assay Kit (Beyotime, China). Briefly, mBMSCs were seeded in 24‐well plates and subjected to the indicated treatments. After treatment, cells were washed with PBS and incubated with DCFH‐DA working solution or MitoSOX Red working solution (5 µM) at 37°C for 30 min in the dark. Nuclei were counterstained with Hoechst 33342 (Yeasen, China). Subsequently, cells were washed three times with PBS to remove excess probe. Cells were observed using a fluorescence microscope. Images were captured under identical acquisition parameters, and fluorescence intensity was quantified using ImageJ software.

Cellular ATP production was measured using a commercial ATP detection kit (Beyotime, China) according to the manufacturer's instructions. Briefly, treated mBMSCs were lysed using the lysis buffer provided in the kit. The lysates were then mixed with the luciferase‐based ATP detection reagent, and luminescence intensity was measured using a microplate luminometer. The ATP concentration was calculated based on a standard curve and normalized to the total protein content.

Mitochondrial membrane potential was evaluated using a JC‐1 mitochondrial membrane potential assay kit (Beyotime, China) according to the manufacturer's instructions. Briefly, mBMSCs were seeded in appropriate culture plates and subjected to the indicated treatments. After treatment, cells were incubated with JC‐1 staining working solution at 37°C for 20 min in the dark. Cells were washed twice with JC‐1 staining buffer to remove excess dye. Fluorescence images were acquired using a fluorescence microscope under identical acquisition parameters. JC‐1 aggregates formed in mitochondria with high membrane potential exhibited red fluorescence, whereas JC‐1 monomers in mitochondria with reduced membrane potential showed green fluorescence. Fluorescence intensity profile analysis was assessed by using ImageJ software.

### 4.9 Mitochondrial Morphology Observation

Mitochondrial morphology in mBMSCs was visualized using Mito‐Tracker Red staining (Beyotime, China). Cells were incubated with the dye at 37°C for the recommended duration, followed by washing with PBS to remove excess dye. Stained cells were observed under a confocal laser scanning microscope. Quantitative analysis of mitochondrial morphology, including mitochondrial number, branch number, average branch length, and mitochondrial network area, was performed using ImageJ software with the Mitochondrial Network Analysis (MiNA) plugin, according to the method described by Valente et al. [75]. Images were subjected to unsharp mask and enhance local contrast, followed by conversion into binary images and skeletonization for quantitative mitochondrial network analysis. Mitochondrial morphological parameters, including mitochondrial number, branch number, mean branch length, and mitochondrial footprint, were quantified using the MiNA analysis workflow.

Transmission electron microscopy (TEM) was used to examine mitochondrial ultrastructure. Cells were fixed with 2.5% glutaraldehyde at 4°C overnight and post‐fixed with 1% osmium tetroxide. Samples were subsequently dehydrated through a graded ethanol series and embedded in epoxy resin. Ultrathin sections were prepared using an ultramicrotome and stained with uranyl acetate and lead citrate. The sections were then observed under a transmission electron microscope (HITACHI, Japan) to evaluate mitochondrial morphology and structural integrity. Mitochondrial morphology in TEM images was quantitatively analyzed using ImageJ software. Multiple mitochondria from randomly selected fields of independent samples were included in the analysis. The major and minor axes of individual mitochondria were measured, and the aspect ratio was calculated as the ratio of the major axis to the minor axis.

### 4.10 Quantitative Real‐Time PCR (RT‐qPCR)

Total RNA was extracted from mBMSCs using TRIzol reagent (Invitrogen, China) according to the manufacturer's instructions. The concentration and purity of RNA were determined using a spectrophotometer. Complementary DNA (cDNA) was synthesized using a reverse transcription kit (Yeasen, China). Quantitative real‐time PCR was performed using SYBR Green Master Mix (Yeasen, China) on a real‐time PCR system. Gene expression levels were normalized to the housekeeping gene and relative expression levels were calculated using the 2^−ΔΔCt^ method. The specific primer sequences used in this study are listed in Supplementary materials (Table S1).

### 4.11 Western Blot Analysis

Total protein was extracted from cells using RIPA lysis buffer containing protease and phosphatase inhibitors. Protein concentrations were determined using a BCA protein assay kit (Beyotime, China). Equal amounts of protein samples were separated by SDS‐PAGE and transferred onto PVDF membranes. After blocking with 5% nonfat milk, the membranes were incubated with primary antibodies against target proteins at 4°C overnight, followed by incubation with appropriate horseradish peroxidase‐conjugated secondary antibodies (CST, America). Protein bands were visualized using an enhanced chemiluminescence (ECL) detection system (Yeasen, China) and quantified by gray value analysis through ImageJ.

### 4.12 Immunofluorescence Staining

Cells were washed three times with PBS and fixed with 4% paraformaldehyde for 20 min at room temperature. After fixation, cells were permeabilized with 0.5% Triton X‐100 for 20 min and blocked with immunofluorescence blocking solution for 2 h at room temperature. Cells were then incubated with primary antibody against the osteogenic marker OPN overnight at 4°C in a humidified chamber. After washing with PBS, cells were incubated with fluorescently labeled secondary antibody for 1 h at room temperature in the dark. Cell nuclei and cytoskeleton were counterstained with DAPI and fluorescent cytoskeletal staining solution, respectively. Images were acquired using a fluorescence microscope under appropriate excitation conditions, and 3D reconstruction of fluorescence intensity maps was analyzed through ImageJ.

### 4.13 RNA Sequencing

RNA sequencing was performed by Guangke Ande Biotechnology Co., Ltd. (Hangzhou, China). Briefly, OP‐mBMSCs treated with GelMA or Mn‐ZIF‐8@GelMA were collected, and total RNA was extracted using TRIzol reagent. RNA quality and concentration were assessed before library preparation. mRNA was enriched using oligo(dT)‐conjugated magnetic beads and used for cDNA library construction. After library quality assessment, sequencing was performed on an Illumina high‐throughput sequencing platform.

Differentially expressed genes (DEGs) were identified using the criteria of |fold change| > 1.3 and p < 0.05. Gene Ontology (GO) enrichment analysis, including biological process (BP), molecular function (MF), and cellular component (CC), and Kyoto Encyclopedia of Genes and Genomes (KEGG) pathway enrichment analysis were performed using the clusterProfiler R package.

### 4.14 Lentiviral Transduction

The shRNA sequence targeting MAVS was cloned into a lentiviral vector carrying GFP fluorescence and puromycin‑resistance genes to construct the MAVS‑knockdown lentiviral vector (shMAVS). An empty lentiviral vector (shNC) was constructed in parallel and served as the negative control. OP‐mBMSCs were seeded in 24‑well plates at a density of 8 × 10^4^ cells per well and cultured overnight to reach approximately 70 % confluency. A half‑volume infection method was adopted, and lentivirus was added to the cells at a multiplicity of infection (MOI) of 30. Virus‑containing medium was removed and replaced with fresh complete medium 24 h after infection for further cultivation. At 72 h post‑infection, cells were cultured in medium containing 7 µg/mL puromycin for screening. The puromycin‑containing medium was refreshed every 2–3 days for 10 days. The surviving cells represented stable cell lines expressing shMAVS or shNC. GFP fluorescence intensity was observed under a fluorescence microscope and Western Blot was performed to detect MAVS protein levels to verify the knockdown efficiency.

### 4.15 In Vivo Extraction Socket Repair Model

To evaluate the regenerative efficacy of Mn‐ZIF‐8@GelMA in vivo, osteoporotic mice were used to establish a maxillary first molar extraction socket model. OVX‐induced osteoporotic mice were randomly divided into three groups: Blank, GelMA, and Mn‐ZIF‐8@GelMA, with five mice in each group. Under anesthesia, the maxillary first molar was carefully extracted, and the integrity of the extracted roots was confirmed. For the Blank group, no material was applied to the extraction socket. For the GelMA and Mn‐ZIF‐8@GelMA groups, the corresponding hydrogel precursor was injected into the extraction socket and photocrosslinked in situ according to the established gelation protocol. Fourteen days after surgery, the mice were sacrificed, and the maxillae as well as major organs, including the heart, liver, spleen, lung, and kidney, were harvested and fixed in 4% paraformaldehyde for subsequent analysis.

The harvested maxillae were scanned using micro‐computed tomography to evaluate new bone formation within the extraction sockets. A region of interest was defined within the extraction socket area, and three‐dimensional reconstruction was performed. Quantitative parameters, including bone volume/total volume (BV/TV), trabecular number (Tb.N), trabecular thickness (Tb.Th), and trabecular separation (Tb.Sp), were analyzed to assess the extent and quality of regenerated bone.

After micro‐CT analysis, the maxillary samples were decalcified using an EDTA‐based decalcifying solution, dehydrated, embedded in paraffin, and sectioned together with the surrounding soft tissues. Hematoxylin and eosin (H&E) staining was performed to evaluate the general morphology of regenerated tissue and new bone formation within the extraction sockets. Masson's trichrome staining was performed to evaluate collagen deposition and extracellular matrix organization during socket repair. For systemic biosafety evaluation, paraffin sections of the heart, liver, spleen, lung, and kidney were stained with H&E and examined for inflammatory infiltration, tissue necrosis, or structural abnormalities.

For immunofluorescence staining, deparaffinized tissue sections were subjected to antigen retrieval, permeabilized, and blocked with 5% bovine serum albumin or normal goat serum to reduce nonspecific binding. The sections were then incubated with primary antibodies against MAVS and BMP‐2 at 4°C overnight, followed by incubation with appropriate fluorescent secondary antibodies at room temperature in the dark. Nuclei were counterstained with DAPI, and the sections were mounted with antifade mounting medium. Fluorescence images were captured using a fluorescence microscope.

### 4.16 Statistical Analysis

All experiments were performed at least three times independently. Data are presented as the mean ± standard deviation (SD). Statistical analyses were conducted using GraphPad Prism software. Data distribution and homogeneity of variance were assessed before statistical comparison. Comparisons between two groups were performed using Student's t‐test, while comparisons among multiple groups were conducted using one‐way analysis of variance (ANOVA) followed by appropriate post hoc tests. When the assumptions for parametric tests were not met, appropriate corrected or nonparametric tests were applied. A value of *p* < 0.05 was considered statistically significant.

## Author Contributions

**Xiayue Jin** and **Shengjia Ye** designed the experiments, performed the majority of the experimental work, analyzed the data, prepared the figures, and wrote the original draft. **Yun Yuan** contributed to RNA sequencing analysis and bioinformatic interpretation. **Xueying Yang** assisted with material preparation, characterization, and in vitro experiments. **Jiarui Cui** assisted with animal experiments, micro‐CT analysis, and histological evaluation. **Hongming Zhang** and **Hui Huang** conceived and supervised the study. Hui Huang and Hongming Zhang provided resources, secured funding, supervised the project, and critically revised the manuscript. All authors discussed the results, reviewed the manuscript, and approved the final version.

## Funding

National Natural Science Foundation of China (No.81771105).

## Ethics Approval

All animal experiments were approved by the Animal Ethics Committee of Shanghai Ninth People's Hospital, Shanghai Jiao Tong University School of Medicine (Approval No. SH9H‐2023‐A890‐1), and were conducted in accordance with the institutional guidelines for the care and use of laboratory animals.

## Conflicts of Interest

The authors declare no conflicts of interest.

## Supporting information




**Supporting File**: advs78268‐sup‐0001‐SuppMat.docx.

## Data Availability

The data that support the findings of this study are available within the article and its Supplementary materials. Additional raw data, including the RNA sequencing data generated in this study, are available from the corresponding author upon reasonable request.
