# Supplementary material for: Mn/Zn Bimetallic MOF‐Integrated Hydrogel Alleviates Excessive Mitochondrial Fission in BMSCs to Promote Osteoporotic Jawbone Repair
Source: Adv Sci (Weinh). 2026 Oct 15:e78268. Online ahead of print. doi: 10.1002/advs.78268 (PMC13672688; doi:10.1002/advs.78268)
Supplement: Supplementary file 1 [file ADVS-9999-e78268-s001.docx]

**Supplementary Materials**

**Figure S1. Establishment and validation of the osteoporotic mouse model. (A)** Schematic illustration of the modeling procedure. **(B)** Body weight of the OP group significantly increased 8 weeks after surgery (n=6 each group). **(C)** Micro-CT images of the femur and quantitative analysis of bone parameters revealed a marked reduction in bone mass and deterioration of bone microarchitecture, confirming the successful establishment of the osteoporotic model (n=6 each group). Error bars indicate the mean ± SD. **p < 0.01, ****p < 0.0001

**Figure S2. Effects of osteoporosis on the jawbone. (A)** Micro-CT images and quantitative bone parameter analysis revealed significant bone loss and deterioration of trabecular microarchitecture in the jawbone under osteoporotic conditions (n=6 each group). **(B)** H&E staining showed that bone tissue in the OP group was markedly sparser, with thinner trabeculae and reduced height and width of the alveolar ridge between the first and second molars (n=6 each group). **(C)** Masson’s trichrome staining demonstrated that bone tissue in the Ctrl group consisted predominantly of dense collagen fibers and mature bone matrix, whereas the OP group exhibited reduced new bone formation and sparse collagen fiber deposition (n=6 each group). Error bars indicate the mean ± SD. ****p < 0.0001

**Figure S3. Macroscopic appearance and photocrosslinking behavior of GelMA and Mn-ZIF-8@GelMA hydrogels.** The blank GelMA solution appeared colorless and transparent, whereas the color of the hydrogel gradually turned yellow with increasing Mn-ZIF-8 nanoparticle content. Before UV irradiation, the hydrogel precursor solution flowed freely inside the glass vial, indicating good fluidity and injectability. Upon exposure to UV light, the hydrogel rapidly crosslinked and solidified to form a stable three-dimensional network with good shape stability and adhesion.

**Figure S4. Mechanical properties of Mn-ZIF-8@GelMA with 0.1%, 0.3%, and 0.5% w/v Mn-ZIF-8**. **(A)** Compressive modulus. **(B)** Tensile modulus.

**Figure S5. Cumulative Zn and Mn ion release profiles from 0.3% ZIF-8@GelMA and Mn-ZIF-8@GelMA hydrogels over 21 days.** At the end of degradation, ZIF-8@GelMA released Zn at a concentration of 15570.27 ± 528.78 μg/L, whereas Mn-ZIF-8@GelMA released Zn and Mn at concentrations of 12516.94 ± 233.75 μg/L and 2658.69 ± 30.84 μg/L, respectively (n=3 each group).

**Figure S6. Characterization of intracellular Mn-ZIF-8 nanoparticle uptake in OP-mBMSCs.** Representative TEM images illustrating electron-dense structures corresponding to internalized Mn-ZIF-8 nanoparticles in OP-mBMSCs (yellow dashed circles) following Mn-ZIF-8@GelMA treatment. Scale bar: 500nm.

**Figure S7. Hemostatic performance of the composite hydrogels. (A)** Representative photographs of the mouse liver injury model. **(B)** Quantitative analysis of blood loss in different treatment groups. Application of hydrogels significantly reduced blood loss compared with the blank group (n=3 each group). Error bars indicate the mean ± SD. Error bars indicate the mean ± SD. ****p < 0.0001

**Figure S8. Mn-ZIF-8@GelMA partially rescues the impaired osteogenic differentiation of OP-mBMSCs. (A)** Representative images of ALP staining and ARS staining in Ctrl mBMSCs, OP-mBMSCs, and Mn-ZIF-8@GelMA-treated OP-mBMSCs (n=3 each group). **(B)** Quantification of relative ALP activity (n=3 each group). **(C)** Relative mRNA expression levels of osteogenesis-related genes, including *alpl, runx2, bmp2*, and *col1a1* (n=3 each group)*.* **(D)** Representative WB images of BMP-2 protein and quantitative analysis of gray value (n=3 each group). Error bars indicate the mean ± SD. **p < 0.01, ***p < 0.001, ****p < 0.0001.

**Figure S9 Mn-ZIF-8@GelMA improves early osteogenic differentiation and migration capacity of OP-mBMSCs. (A)** Representative images of ALP staining in OP-mBMSCs treated with or without Mn-ZIF-8@GelMA (n=3 each group). Scale bar: 500 μm. **(B)** Quantitative analysis of relative ALP activity. **(C)** Representative images of wound healing assay at 0 and 48 h and quantitative analysis of wound closure rate (n=3 each group). Scale bar: 500μm. Error bars indicate the mean ± SD. ***p < 0.001, ****p < 0.0001.

**Figure S10. Effects of Mn-ZIF-8@GelMA, NOX inhibition, and mitochondrial-targeted antioxidant treatment on (A) intracellular and (B) mitochondrial ROS in OP-mBMSCs** (n=3 each group)**.** Scale bars: 500 μm.

**Figure S11. Cytocompatibility of OP-mBMSCs treated with ZIF-8@GelMA and Mn²⁺-loaded GelMA.** Cell viability of OP-mBMSCs cultured with no treatment (OP), ZIF-8@GelMA, or Mn²⁺-loaded GelMA was evaluated using the CCK-8 assay at days 1, 3, and 5 (n=3 each group). Error bars indicate the mean ± SD. *p < 0.05, **p < 0.01, ****p < 0.0001.

**Figure S12. Comparison of Mn-ZIF-8@GelMA and ZIF-8@GelMA in regulating osteogenic differentiation, migration, mitochondrial function, and oxidative stress in OP-mBMSCs. (A)** Representative ALP and ARS staining images of OP-mBMSCs treated with Mn-ZIF-8@GelMA or ZIF-8@GelMA (n=3 each group). Scale bars: 500 μm. **(B)** Relative mRNA expression levels of osteogenesis-related genes, including *alpl, runx2, bmp2,* and *col1a1*(n=3 each group). **(C)** Western Blot images of MAVS, p-DRP1, DRP1, and BMP-2 expression (n=3 each group). **(D)** Representative scratch wound assay images and quantitative analysis of wound closure at 48 h (n=3 each group). Scale bars: 500 μm. **(E)** Relative intracellular ATP levels (n=3 each group). **(F)** Representative DCFH-DA fluorescence images showing intracellular ROS levels (n=3 each group). Scale bars: 500 μm. **(G)** Representative MitoSOX fluorescence images showing mitochondrial ROS levels (n=3 each group). Scale bars: 500 μm. Error bars indicate the mean ± SD. *p < 0.05, **p < 0.01, ***p < 0.001, ****p < 0.0001.

**Figure S13. Expression of MAVS, DRP1, phosphorylated DRP1 (p-DRP1), and BMP-2 in control and OP mBMSCs** (n=3 each group)**.**

**Figure S14. Schematic diagram of in vivo experimental design for maxillary first molar extraction in osteoporotic mice.** Groups: No treatment post-extraction (Blank group), immediate GelMA application post-extraction (GelMA group), and immediate Mn-ZIF-8@GelMA application post-extraction (Mn-ZIF-8@GelMA group).

**Figure S15. H&E staining of major organs 14 days after tooth extraction in the mouse upper first molar extraction model** (n=5 each group)**.**

**Table S1.** Primers used for RT-qPCR in this study.

| Genes | Forward（5’-3’） | Reverse（3’-5’） |
| --- | --- | --- |
| *alpl* | CCAACTCTTTTGTGCCAGAGA | TGACATTCTTGGCTACATTGGTG |
| *runx2* | GACTGTGGTTACCGTCATGGC | ACTTGGTTTTTCATAACAGCGGA |
| *bmp2* | GGGACCCGCTGTCTTCTAGT | TCAACTCAAATTCGCTGAGGAC |
| *col1a1* | GCTCCTCTTAGGGGCCACT | ATTGGGGACCCTTAGGCCAT |
| *sirt1* | TGATTGGCACCGATCCTCG | CCACAGCGTCATATCATCCAG |
| *nrf2* | CTTTAGTCAGCGACAGAAGGAC | AGGCATCTTGTTTGGGAATGTG |
| *fis1* | CCGGCTCAAGGAATATGAAA | ACAGCCAGTCCAATGAGTCC |
| *opa1* | ATACTGGGATCTGCTGTTGG | AAGTCAGGCACAATCCACTT |
| *mfn1* | ATGGCAGAAACGGTATCTCCA | GCCCTCAGTAACAAACTCCAGT |
| *mfn2* | ATGTTACCACGGAGCTGGAC | AACTGCTTCTCCGTCTGCAT |
| *dnm1l* | TGCAGCTAGTCCACGTTTCA | CCCATTCTTCTGCTTCAACTCT |
| *lgfbp4* | AGAAGCCCCTGCGTACATTG | TGTCCCCACGATCTTCATCTT |
| *ccnd1* | GCGTACCCTGACACCAATCTC | CTCCTCTTCGCACTTCTGCTC |
| *tubb5* | GATCGGTGCTAAGTTCTGGGA | AGGGACATACTTGCCACCTGT |
| *itga6* | TGCAGAGGGCGAACAGAAC | GCACACGTCACCACTTTGC |
| *spp1* | AGCAAGAAACTCTTCCAAGCAA | GTGAGATTCGTCAGATTCATCCG |
| *slc25a5* | CAAGACAGCGGTAGCACCC | CGCAGTCTATGATGCCCTTGTA |
| *cd44* | TCGATTTGAATGTAACCTGCCG | CAGTCCGGGAGATACTGTAGC |
| *rrm2* | TGGCTGACAAGGAGAACACG | AGGCGCTTTACTTTCCAGCTC |
| *fosl2* | CCAGCAGAAGTTCCGGGTAG | GTAGGGATGTGAGCGTGGATA |
| *tgfbr3* | GGTGTGAACTGTCACCGATCA | GTTTAGGATGTGAACCTCCCTTG |
| *tsc22d1* | CCAGTGGCGATGGATCTAGGA | CTTGCACCAGAGCTATTGTCA |
| *selenop* | AGCTCTGCTTGTTACAAAGCC | CAGGTCTTCCAATCTGGATGC |
| *sesn3* | CGGAAGGACAAAAGAATCCGA | GTTCATCCGCCGTATTTGCT |
| *osmr* | CATCCCGAAGCGAAGTCTTGG | GGCTGGGACAGTCCATTCTAAA |
| *hmga1* | GGTCGGGAGTCAGAAAGAGC | ATTCTTGCTTCCCTTTGGTCG |
| *mavs* | CTGCCTCACAGCTAGTGACC | CCGGCGCTGGAGATTATTG |
| *b-actin* | AGAAGATCTGGCACCACACC | TACGACCAGAGGCATACAGG |
